# Discovery of mono-ADP ribosylating toxins with high structural homology to *Pseudomonas* exotoxin A

**DOI:** 10.1038/s42003-025-07845-y

**Published:** 2025-03-11

**Authors:** Geoffrey Masuyer, Alistair Taverner, Julia MacKay, Ana Rita Lima Marques, Yuye Wang, Tom Hunter, Keyi Liu, Randall J. Mrsny

**Affiliations:** 1 Department of Biochemistry and Biophysics, Stockholm University, Stockholm, Sweden; 2 Department of Life Sciences, University of Bath, Bath, UK; 3 Centre for Therapeutic Innovation, University of Bath, Bath, UK; 4 Applied Molecular Transport Inc., South San Francisco, CA USA

**Keywords:** Proteins, X-ray crystallography, Pathogens

## Abstract

Mono-ADP-ribosyl transferase (mART) proteins are secreted virulence factors produced by several human pathogens, the founding member being diphtheria toxin (DT). *Pseudomonas aeruginosa* can also secrete a mART toxin, known as exotoxin A (PE), but with an organization of its three functional domains (receptor, translocation, and enzymatic elements) that is opposite to DT. Two additional PE-like toxins (PLTs) have been identified from *Vibrio cholerae* and *Aeromonas hydrophila*, suggesting more PLT family members may exist. Database mining discovered six additional putative homologues, considerably extending this group of PLTs across a wide range of bacterial species. Here, we examine sequence and structural information for these new family members with respect to previously identified PLTs. The X-ray crystal structures of four new homologues show the conservation of critical features responsible for structure and function. This study shows the potential of these newly described toxins for the development of novel drug delivery platforms. Additionally, genomic analysis suggests horizontal gene transfer to account for the wide distribution of PLTs across a range of eubacteria species, highlighting the need to monitor emerging pathogens and their virulence factors.

## Abstract (web summary)

Mono-ADP-ribosyl transferase (mART) proteins, including exotoxin A (PE) from Pseudomonas aeruginosa are a family of virulence factors produced in various bacteria that promote pathogenesis. This study identifies new homologues through database mining and structural analysis, highlighting their potential for drug delivery applications and the role of horizontal gene transfer in their widespread distribution.

## Introduction

Bacterial-derived mono-ADP-ribosyl transferase (mART) proteins are a class of exceptionally potent virulence factors, with diphtheria toxin (DT) secreted by *Corynebacterium diphtheriae* being the first identified example^1^. Proteins secreted by *Pseudomonas aeruginosa* (PE)^2^, *Vibrio cholerae* (Chx)^3^, and *Aeromonas hydrophila* (Ahx)^4^ have expanded this family. All four of these A–B-type toxins are single-chain proteins that fold into three spatially distinct structures that appear to function independently for receptor-mediated endocytosis, translocation to the host cell cytoplasm, and catalytic ADP-ribosylation activity. These toxins specifically target the diphthamide residue of eukaryotic ribosomal elongation factor 2 (eEF2), an enzyme that is essential for protein synthesis^5^. Transfer of ADP-ribose to the diphthamide from nicotinamide adenine dinucleotide (NAD + ), directly inhibits eEF2 and leads to cell death^6^. DT is organized with the catalytic activity at the N-terminus and the endocytosis element at the C-terminus of the protein but exotoxins from *Pseudomonas*, *Vibrio*, and *Aeromonas* present a reverse architecture. Differences in functional domain organization imply distinct intracellular trafficking fates. PE and Chx bind to LRP1 (low-density lipoprotein receptor-related protein 1) at the cell surface, initiating receptor-mediated endocytosis^3,7^. Subsequently, these toxins are transported in a retrograde manner to the endoplasmic reticulum (ER) in a mechanism involving a C-terminal target sequence that is not present in DT^8^, during which they are processed by furin, a host proprotein convertase. This enzymatic cleavage converts the toxins into di-chain molecules linked by a single disulphide bond. The carboxy-terminal fragment, containing the catalytic domain, is then released and translocated into the cytosol^9^, where it catalyses ADP-ribosylation of eEF2^10^. Such organizational differences between DT-like toxins (DLTs) and *Pseudomonas*-like toxins (PLTs) suggest distinct virulence strategies taken by pathogens that may be more widespread than the currently identified proteins. Indeed, additional DLTs have recently been identified in several *Austwickia*, *Seinonella*, and *Streptomyces* species of bacteria^11,12^. These toxins present strong structural conservation to DT but showed variations in their individual domain’s activity that likely reflect how they have adapted to their target host^13^. Remarkably, these properties have proved useful for the development of novel drug delivery tools^14^.

We performed a search of public databases using selected hallmarks present in the amino acid sequences of the three known PLTs to identify six additional family members and performed structural and functional assessments of all nine family members. These additional six PLTs were identified across a diverse spectrum of bacterial species: *Chromobacterium haemolyticum, Janthinobacterium lividum*, *Collimonas fungivorans*, *Serratia fonticola*, *Acinetobacter baumannii*, and *Shewanella putrefaciens*. Due to the expanded nature of this family of proteins, we propose a set of three-letter abbreviations as a more consistent nomenclature used for these comparisons, with a historical perspective of PE and Chx. All these PLTs were composed of a single-chain amino acids that folded into three distinct structures: a receptor binding domain I, a translocation domain II, and a catalytic toxin domain III. In vitro, functional assessment of specific domain properties demonstrates the potential impact of sequence variations on the functional properties of these PLTs.

X-ray crystallographic comparisons demonstrated nearly superimposable structures for all PLTs. The broad range of bacteria now identified to express this type of exotoxin suggests a selective advantage for these proteins in a variety of environmental and/or host situations, and raises the question of how this protein function was acquired across these diverse species. Phylogenetic analysis suggested a bias toward horizontal gene transfer (HGT) for genetic distribution that can be followed through a dissemination hierarchy. In sum, our studies have provided evidence of a dramatically expanded set of PLTs with highly similar structures, that have disseminated across a broad range of bacterial species. This information expands our understanding of the potential role of PLTs with possible clinical, pharmaceutical, and environmental impact, extending the potential for biotechnological applications of these proteins.

## Results

### Identification and overall structure of PLT proteins

A public database search using the amino acid sequence of *Pseudomonas aeruginosa* exotoxin A protein (UniProt entry P11439) as a template for specific features was carried out against the GenBank database with the BLASTP tool, where entries from *Pseudomonas*, *Vibrio*, and synthetic constructs were excluded. Only those proteins noted above with homology criteria spanning across the full length of the toxin (>88%) were selected for analysis. Beyond this group, several distant homologues with partial sequence coverage (6–33%) were detected, and although some showed notable identity values and potentially significant E-values (9e^−89^ to 2e^−05^), the homology was only related to the enzymatic domain, and they were excluded as potential PLTs. Domain conservation was confirmed using Prosite and InterProScan. This search strategy led to the discovery of the recently described *Aeromonas hydrophila* exotoxin A (Ahx)^4^, and PLTs from six other bacterial species: *Acinetobacter baumannii, Chromobacterium haemolyticum, Collimonas fungivorans*, *Janthinobacterium lividum, Shewanella putrefaciens*, and *Serratia fonticola*. Some physical details about these PLTs and proposed abbreviations for each PLT family member are shown in Supplementary Table 1. These abbreviations use the well-established nomenclature for exotoxins from *Pseudomonas aeruginosa* and *Vibrio cholerae* but use genus and species names to guide the remaining aspect of this nomenclature, with the caveat of *Chromobacterium haemolyticum* where there would be conflict with the *Vibrio cholerae* toxin name. Of note, the GC content of the genes encoding these PTLs varied from 43% to 68%, consistent with a wide distribution of the species identified. All proteins were within a predicted molecular weight (MW) range from 66,596 Da to 73,057 Da and with calculated isoelectric point (pI) values over a range from 5.00 to 6.77.

**Fig. 1:**
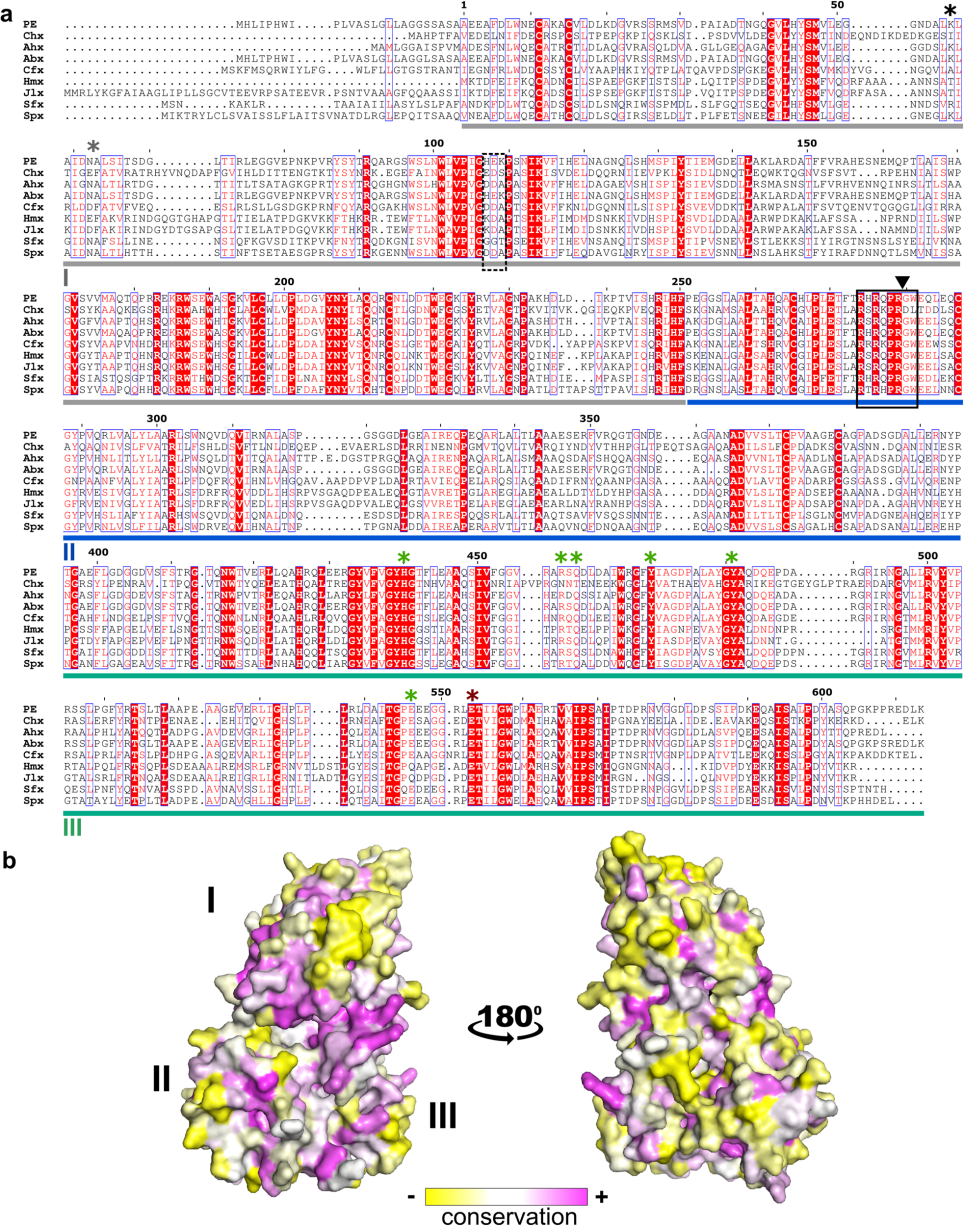
Comparison of PLT family member proteins. **a** Amino acid sequence alignment, showing strictly conserved residues in red shadowing and white text and similar residue types displayed in red text with similar groups of residues boxed in blue. Sequence numbering is based on previous PE descriptions omitting the N-terminal signal peptide^2^, and starting with the mature toxin. Domains I, II, and III are underlined in grey, blue, and green, respectively. The furin cleavage site is indicated with a black triangle, and the consensus furin recognition motif is boxed in black, with the potential secondary site (‘*loop I*’) in the dotted line. Residues from PE identified to interact with the LRP1 receptor are indicated with black (K57) and grey (positions 60–61) stars. Conserved residues for NAD binding and catalytic activity are indicated with green stars, with the key glutamate active site residue in red (E553). Accession numbers for each PLT from GenBank: PE, AKG00567.1; Chx, QKU75906.1; Ahx, APJ14853.1; Abx, SCY08530.1; Cfx, PFH04378.1; Hmx, OQS34021.1; Jlx, AMC33769.1; Sfx, QIP94533.1; Spx, WP_152829160.1. **b** Global conservation of surface amino acids was calculated based on sequence alignment in (a) and displayed on PE structure. Surface colouration describes the most variable (yellow) to most conserved (purple) residues.

Comparison of amino acid sequences showed moderate similarities between these nine potential PLTs across all three domains, with domain I being the most variable (33.2–58.4% homology with PE) and domain III being the least variable (40.9–72.0% homology with PE) between these proteins (Supplementary Fig. 1). There were two notable exceptions: PE was nearly identical to *Acinetobacter baumannii* exotoxin A (Abx), and proteins identified from *Chromobacterium haemolyticum* protein (Hmx) and *Janthinobacterium lividum* (JIx) showed high similarity (82.9%). Sequence alignments, prepared using ESPript^15^, showed the conservation of key cysteines that provide a method of registering these PLT proteins to each other and highlight criteria used in our database screening strategy for their identification (Fig. 1a). All nine PLTs present several key features anticipated as important for PLT structure and function^16^. The pattern of conserved amino acids was striking in selected areas of all three domains with eight cysteine residues at positions 11, 15, 197, 214, 265, 287, 372, and 379 (using numbering for PE in Fig. 1a) being consistent with the four cysteine bridges, as well as amino acids 276-279 defining a consensus ‘RxPR’ furin cleavage sequence. Amino acids with over 75% homology at a specific location were used to describe a consensus sequence for these PLTs (Fig. 1a). Using the established structure of PE [PDB 1IKQ], we used ConSurf^17^ to map the variability level of amino acids for all nine PLT sequences (Fig. 1b). Surface exposed amino acids were highly conserved in only a few selected sites across all three domains of these proteins, with much of these surface-exposed regions being moderate to highly variable.

**Fig. 2:**
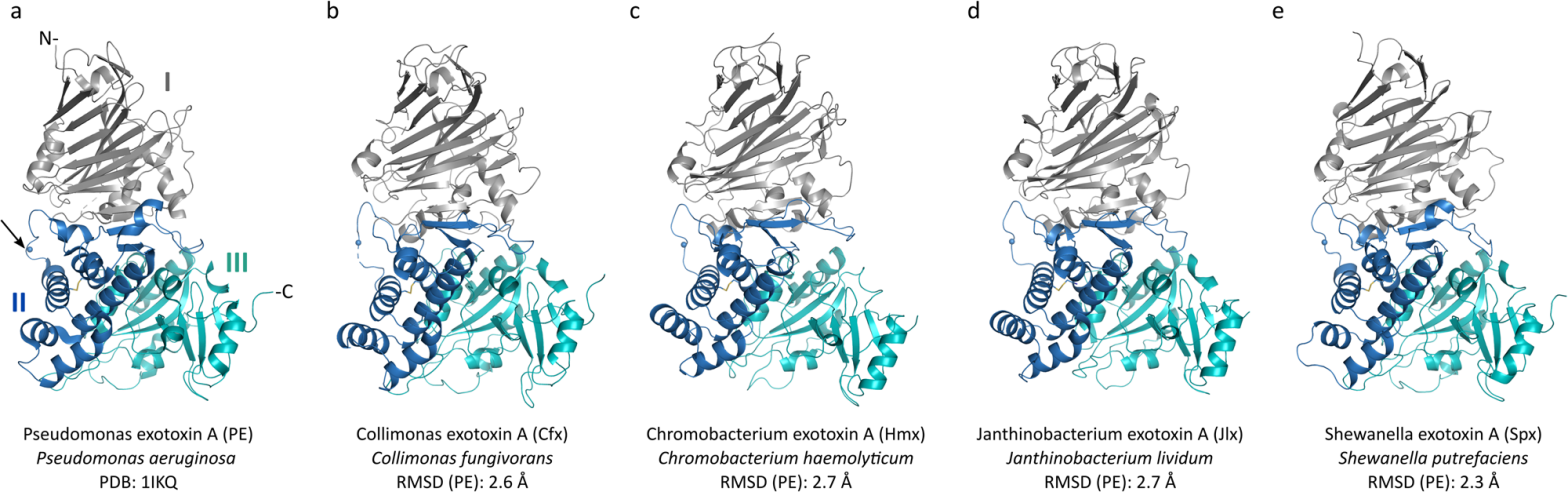
Crystal structure comparison of PLT proteins. **a** PE [PDB 1IKQ], **b ***Collimonas fungivorans* (Cfx), **c ***Chromobacterium haemolyticum* (Hmx), **d ***Janthinobacterium lividum* (Jlx), and **e ***Shewanella putrefaciens* (Spx) exotoxins are shown with domain I in grey, II in blue, and III in cyan. The Furin site in PE is indicated by an arrow pointing to a bead and the equivalent site is represented as a bead in the other structures (**a**–**e**). Overall root mean square deviations (r.m.s.d.) of each structure vs PE are provided (see also Supplementary Table 2).

Despite the high variability and limited consistency of surface-exposed amino acids between PE, Chx, and Ahx, they have highly similar structures^3,4^. The hypothesis that these six newly identified PLTs would also share this structural organization was tested by determining X-ray crystal structures for four of them: *Collimonas fungivorans* (Cfx) to a resolution of 2.8 Å, *Chromobacterium haemolyticum* (Hmx) at 1.35 Å*, Janthinobacterium lividum* (Jlx) at 1.75 Å, and *Shewanella putrefaciens* (Spx) at 1.8 Å (Table 1); a crystallization effort for Abx structure was not performed due to its high amino acid similarity (98%) with PE, and although crystals were obtained for *Serratia fonticola* (Sfx), they did not diffract to a sufficient resolution to solve its structure. Despite having disparate levels of sequence homology, all PLTs had nearly superimposable structures compared to PE (Fig. 2) with pairwise analysis showing root-mean-square deviation (RMSD) values ranging between 2.3 Å and 3.2 Å (all atoms, Supplementary Table 2). All proteins presented a tri-domain architecture like PE, Chx, and Ahx with four strictly conserved disulphide bridges and an exposed loop containing a consensus furin cleavage sequence (Fig. 2). The receptor-binding function is held by domain I^3,7^, which is composed of a core 13-stranded β-jellyroll fold. Since the description of the first PE crystal structure^18^, domain I was proposed to include an additional region, a small segment flanked between domain II and III and labelled ‘domain Ib’ which consists of two β-strands that run anti-parallel to the core domain I. Here we suggest this feature should be considered as an integral component of domain II. Indeed, domain I was shown to fulfil its receptor-binding function on its own^19^, the segment formerly called Ib is, therefore, more likely to be involved in the function held by domain II. Domain II is involved in intracellular trafficking and consists of a compact six α-helices bundle. Importantly, it also presents two conserved key elements that define this family of toxins: the furin recognition site and a disulphide bridge. Proteolytic activation by furin in host target cells is required to convert PLTs into their toxic di-chain mode whilst the adjacent disulphide bridge maintains structural integrity until it is later reduced to promote cytosolic release of the enzymatic domain^9^. The ADP-ribosylating activity of domain III is provided by a unique α/β fold which is specific to this class of toxins. A structural similarity search with domain III using the DALI tool^20^ showed that the catalytic domain of DT is the closest homologue (RMSD of 3.1 Å over 140 residues, 23% sequence identity), followed by several mammalian proteins with lower sequence homology (less than 15%), such as human poly ADP-ribose polymerase 1 (PARP1).

**Table 1:** X-ray crystallography for data collection and refinement statistics of new PLTs

	Cfx	Hmx	Jlx	Spx
**Data collection**				
Beamline	DLS-I03	DLS-I04	DLS-I04	DLS-I04
Wavelength (Å)	0.9763	0.9795	0.9793	0.9795
Space group	P6_2_22	P 2_1_2_1_2_1_	C 121	P 3_1_
Cell dimensions:				
*a, b, c* (Å)	141.8, 141.8, 202.7	66.3, 76.0, 130.5	224.6, 143.2, 110.7	121.0, 121.0, 125.3
α, β, γ (°)	90.0, 90.0, 120.0	90.0, 90.0, 90.0	90.0, 119.4, 90.0	90.0, 90.0, 120.0
Resolution (Å)	70.9–2.80 (2.95–2.80)^a^	65.7–1.35 (1.37–1.35)^a^	115.6–1.75 (1.95–1.75)^a^	104.8–1.79 (1.82–1.79)^a^
No. total/unique reflections	1,201,539/30,359	1,698,060/143,804	2,627,243/186,952	4,151,385/193,517
*R*_meas_	0.505 (5.88)^a^	0.067 (1.86)^a^	0.103 (2.15)^a^	0.287 (3.78)^a^
*R*_pim_	0.109 (1.26)^a^	0.026 (1.01)^a^	0.028 (0.558)^a^	0.062 (0.811)^a^
CC_1/2_	0.998 (0.439)^a^	1.00 (0.437)^a^	1.00 (0.722)^a^	0.997 (0.339)^a^
<*I*/σ(*I*)>	7.6 (0.9)^a^	16.3 (0.7)^a^	12.5 (1.2)^a^	7.5 (0.4)^a^
Completeness (%)	100 (100)^a^	99.1 (90.8)^a^	94.6 (71.3)^a^	100 (99.7)^a^
Redundancy	39.6 (41.2)^a^	11.8 (5.7)^a^	14.1 (14.7)^a^	21.5 (21.2)^a^
**Refinement**				
*R*_work_/*R*_free_	0.237/0.259	0.139/0.173	0.213/0.247	0.185/0.220
**B-factors (A**^b^**)**				
Protein (all atoms)^b^	86.6	23.7	31.7/33.4/33.7/32.9	36.0/37.5/35.2
Solvent	54.8	37.2	32.7	39.6
R.m.s.d. Bond lengths (Å)	0.005	0.008	0.006	0.005
R.m.s.d. Bond angles (°)	1.40	1.45	1.35	1.28
**Ramachandran statistics**				
Favoured (%)	92.2	99.0	97.2	97.2
Outliers (%)	1.8	0.00	0.20	0.11
Clashscore^c^	4.29	4.31	3.72	2.35
**PDB ID**	9G7M	9G7N	9G7O	9G7P

^a^Values in parentheses are for the highest-resolution shell.

^b^Values for each molecule of the asymmetric unit.

^c^Value for all atoms, calculated with MolProbity.

### Domain I

**Fig. 3:**
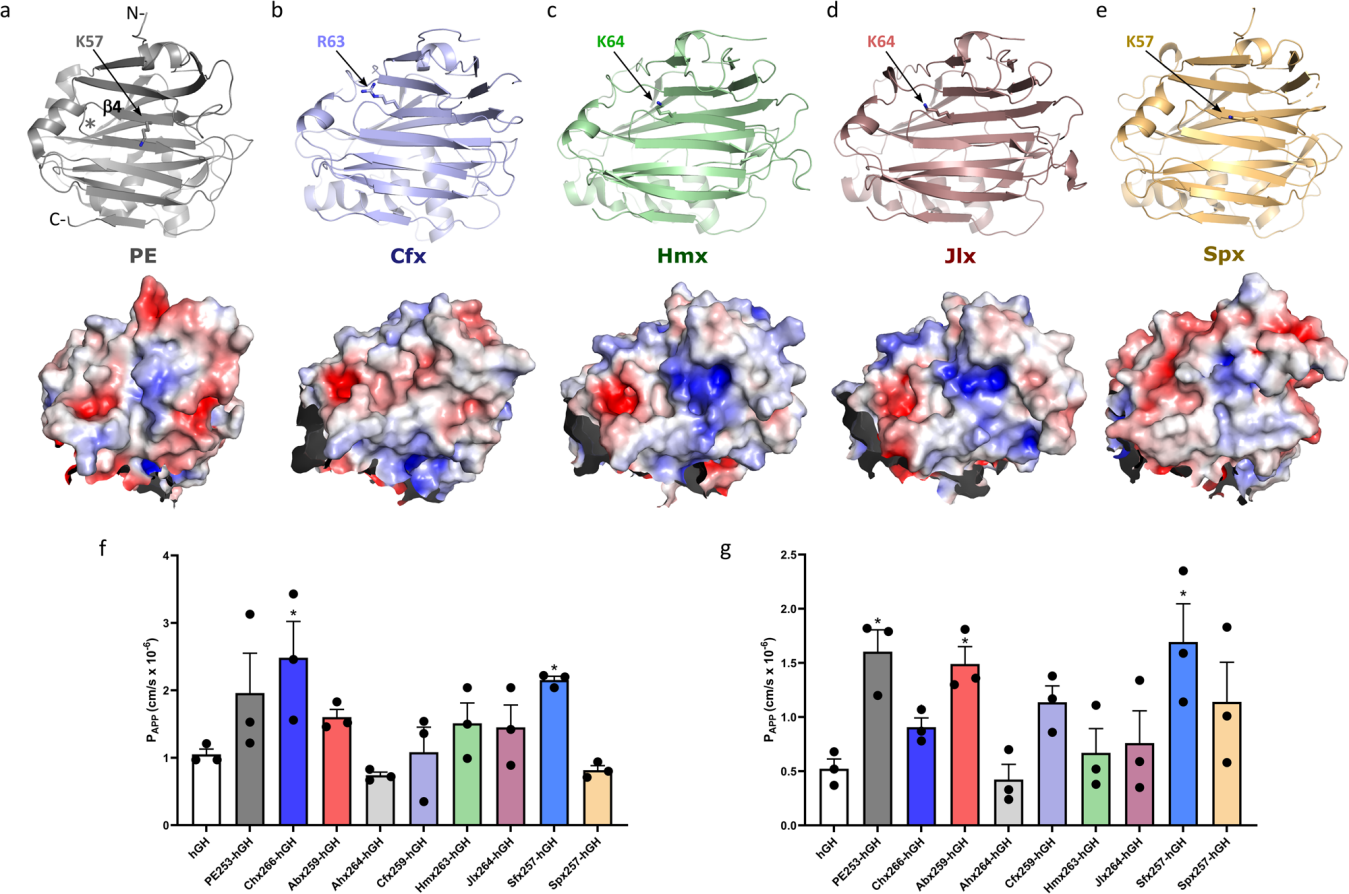
Structure of domain I and transepithelial transport of PLTs. **a** PE (PDB 1IKQ; grey), **b ***Collimonas* (Cfx; blue), **c ***Chromobacterium* (Hmx; green), **d ***Janthinobacterium* (Jlx; red), and **e ***Shewanella* (Spx; yellow) exotoxins in upper panels. The lower panels show the electrostatic surface potential of domain I from each toxin, calculated using the APBS tool in PyMOL (scale from -5, red to +5, blue), with proteins in the same orientation as the upper panels. **f**, **g** Fusion proteins composed of domain I regions of each PLT genetically conjoined to human growth hormone (hGH) were examined for their capacity for apical to basal transport across human (**f)** small intestinal (SMI-100) or (**g)** airway (AIR-100) cell monolayers. Domain I PLT-hGH fusions were applied to apical surfaces at 20 µg/mL with total transport determined after 2 h by ELISA for hGH with standard curves prepared using each chimera. *n* = 3; mean ± SEM. A one-way ANOVA test was used and showed there was a significant variance between the data sets, with ^*^p < 0.05 compared to the control group in a Bonferroni post-test. The *p*-values for (**f**) are overall ANOVA *p* = 0.0118. Chx *p* = 0.0301. Sfx *p* = 0.0234, and (**g)** overall ANOVA *p* = 0.0052. PE *p* = 0.0217. Abx *p* = 0.0457. Sfx *p* = 0.0118.

Domain I, denoted by the grey underline in Fig. 1a, displays a core structure that resembles a fold associated with carbohydrate-binding in β-sandwich type lectins^21^. There is however no current evidence that PE or its homologues interact with glycans. Although this domain presents more sequence variation than in the rest of these toxins (Supplementary Fig. 1, Supplementary Table 2), the domain I core structure is remarkably similar across all toxins with RMSD values between 2.0 Å and 2.5 Å, with the most noticeable differences being observed in the length and positioning of the connecting loops (Fig. 3). As demonstrated for PE and Chx, Domain I has an essential transport function, facilitating receptor-mediated endocytosis (RME) to enable subsequent intracellular trafficking and transcytosis of the PLT protein across polarized epithelial cells in an intact form. Following this apical to basal (A → B) vesicular transcytosis, domain I subsequently mediate RME events that result in a different intracellular trafficking step and delivery of the mART activity to the cytoplasm of non-polarized cells present in the lamina propria^22^. The cell surface receptor identified for PE, and suggested for Chx, is LRP1 (low-density lipoprotein receptor-related protein 1)^3,7^. The ligand-binding activity of LRP1 is mostly driven by its α-chain, which includes four clusters of complement-like repeats and EGF-like domains. The interaction with PE was narrowed down to cluster IV of LRP1, a region close to the cell membrane which is recognised by PE through an interaction mediated by K57 (Fig. 3). Mutation of this lysine in PE was shown to dramatically reduce its cytotoxicity and cell binding ability^19^, whilst insertion of an ‘EF’ dipeptide in the adjacent loop (position 60–61) also affected receptor binding^23^. Primary sequence alignment of PLT proteins indicates that K57 is conserved in Spx but not in the others. However, superposition of the crystal structures shows the presence of a comparatively positioned and positively charged lysine or arginine residue on the same β-strand (β4) in Cfx, Hmx, and Jlx. Interestingly the short downstream loop (‘loop 60’) is also similar across all the toxins, supporting a possible role for that area of domain I in receptor binding events (Fig. 3). As K57 has been suggested to be important in RME and epithelial transcytosis of PE^19^, and since Chx lacks such a charge in β4 of domain I, it is notable that TMEM132A appears to be involved in epithelial transcytosis of Chx^24^.

We directly tested the potential for domain I to function in A → B epithelial transcytosis of these PLT protein family members by testing for the ability to efficiently transport across confluent monolayers of human small intestinal (SMI-100) and airway (AIR-100) epithelia in vitro (Fig. 3b). Apparent permeability (P_APP_) is a widely used measure of the rate at which a molecule can cross an epithelial barrier in vitro. P_APP_ was calculated for the transport of each PLT across small intestinal (SMI-100) and airway (AIR-100) models of human epithelia. The domain I sequence of each PLT protein was genetically fused at its C-terminus to the N-terminus of human growth hormone (hGH), allowing hGH to be the control for non-specific uptake as previously described, and for direct comparison of A → B vesicular transcytosis capacity^22^. While domain I of most PLTs enhanced hGH transport in a manner consistent with RME and vesicular trafficking, culminating in A → B transcytosis as previously observed from PE and Chx^24,25^, they were not equivalent. Chx_266_hGH, Abx_259_hGH, and Sfx_257_hGH, were the most efficient in transporting hGH across SMI-100 monolayers (Fig. 3f), while PE_259_hGH, Chx_266_hGH, Abx_259_hGH, Cfx_259_hGH, and Sfx_264_hGH were superior to the other PLTs for transporting hGH across AIR-100 monolayers (Fig. 3g). These data, delivering a conjoined hGH protein, suggest that any bias of transport outcomes due to differences in protein folding was unlikely and that PLT amino acid sequences in domain I are potentially tuned toward specific host targets. At this point, it is unknown if transport differences measured in these studies were due to RME and/or transcytosis events. Another aspect to consider is that domain I is also responsible for RME into non-polarized cells with a trafficking outcome distinct to A → B transcytosis; it is the composite of interactions with both polarized and/or non-polarized cells that would dictate potential pathogen tropism and the possible role played by these PLTs during an infection. Differences observed for transcytosis efficiency of PLT domain I-hGH chimeras is consistent with this concept.

### Domain II

Domain II of these PLTs, denoted by the blue underline in Fig. 1a, showed a highly conserved organization consisting of a six α-helices bundle followed by two β-strands (formerly described as domain Ib) that sits at the centre of the toxin and complements the domain I core β-sandwich leading into domain III (Fig. 2). Interestingly, a search on the DALI server^20^ for structural homologues of domain II shows that the closest folds only present limited similarity (*Z*-score ≤ 5) and seem to be mostly involved in DNA-binding, such as the single chain Integration Host Factor protein (scIHF2; PDB 2IIE, with a *Z*-score of 5.1 and RMSD of 3.0 Å over 70 aligned residues) or the chromosomal replication initiator protein dnaA (PDB 1L8Q with a *Z*-score of 4.8 and RMSD of 3.5 Å over 72 aligned residues). This region of PE is critical for its secretion from the bacterium^26^, and for cell membrane translocation of the furin-processed portion of the toxin that reaches the host cytoplasm as a trafficking outcome in non-polarized cells, following RME^27^. At present, molecular details of these events are not fully understood so it is unclear if these are distinct or related processes, with one occurring in the bacterium and the other in a host cell. Endoproteolytic activation of PE by furin into a di-chain fragment is necessary for cytoplasmic delivery of the ADP ribosylating activity^9^. Following furin cleavage, the catalytic C-terminal region remains associated with the N-terminal region via a conserved disulphide bridge that is reduced downstream of the endocytic pathway with the help of protein disulphide-isomerase^28^. Furin is ubiquitously expressed as a type-I transmembrane protein present in various cell compartments and involved in intracellular endosomal trafficking and the trans-Golgi network^29^. In the case of PE, furin cleavage occurs preferentially at acidic pH in vitro, reflecting endosome conditions (pH < 5.5) in a non-polarized host cell where cleavage is believed to occur in vivo^30^. The furin consensus sequence RxPR was strictly conserved in all PLTs, starting at position 276 of PE with x in this sequence being restricted to the amino acids K, Q, or H across the family members (Fig. 1a).

**Fig. 4:**
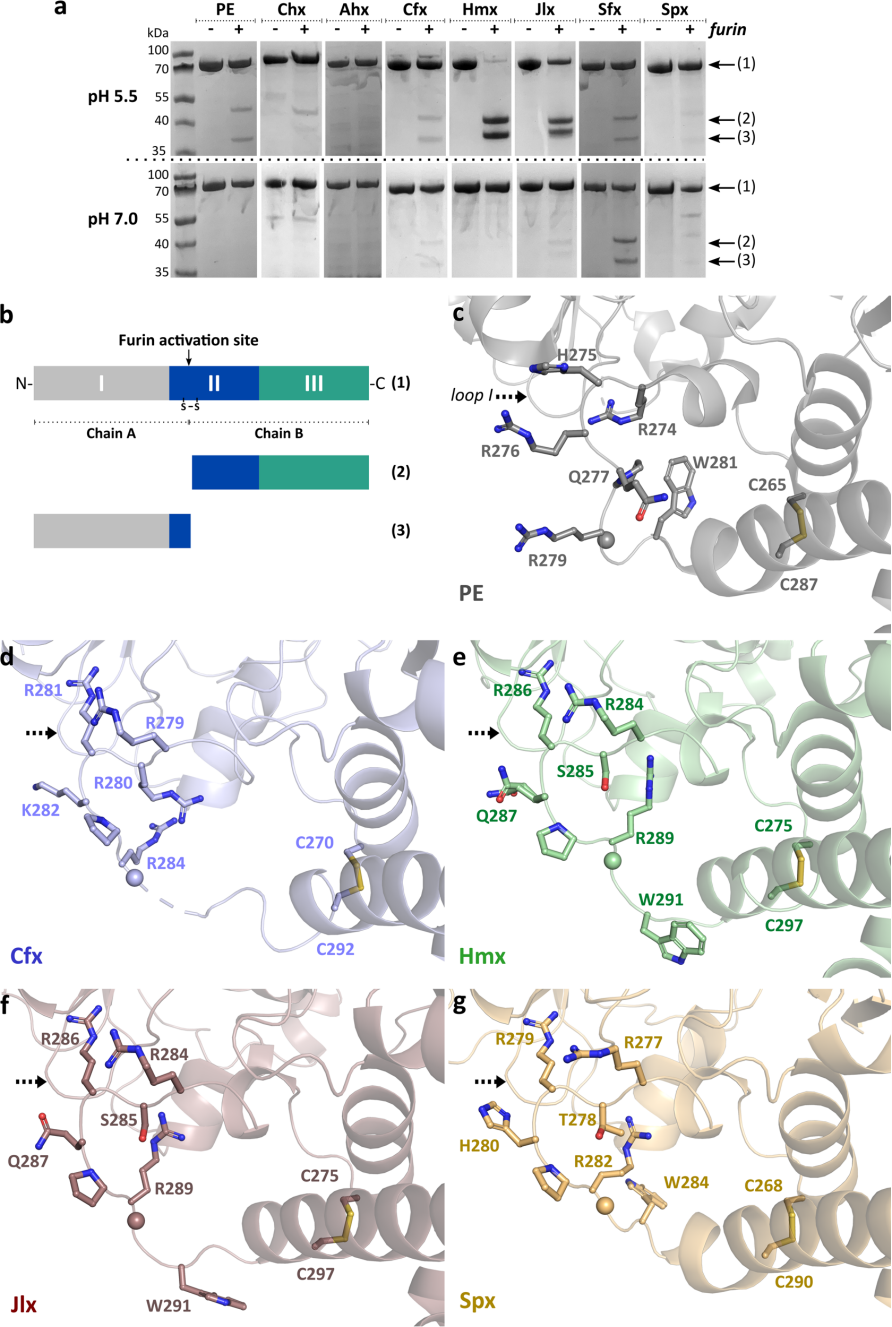
Furin activation loop. **a** Activation of PLTs by furin. Proteins were incubated with (+) or without (−) furin for 1 h at 25 °C, and loaded onto an SDS-PAGE gel in a reducing buffer. Corresponding elements are labelled as described in (**b**). Variations in the fragment sizes follow the difference in the size of each PLT ranging from 66.6 to 73.0 kDa before cleavage (Supplementary Table 1 and Fig. 2). **b** Schematic of the PE structure showing the di-chain component resulting from furin cleavage. **c** Structure of PE (PDB 1IKQ; grey) with the conserved furin recognition site shown as sticks and scissile bond as a sphere. The loop (labelled ‘loop I’) with strong sequence variation within proximity of the furin cleavage site is indicated. Structures of **d ***Collimonas* (Cfx; blue), **e ***Chromobacterium* (Hmx; green), **f ***Janthinobacterium* (Jlx; red), and **g ***Shewanella* (Spx; yellow) exotoxins, labelled as per (**c**) with disordered Cfx region in (**d**) marked by a dashed line.

Previous studies suggested that PE might undergo conformational changes at acidic pH, which could in turn promote its presentation to furin^30,31^. Interestingly, crystals of the PLTs described here were obtained over a pH range of 5.5–7.0, hinting that the overall structure is unlikely to change significantly in slightly acidic conditions. Since furin efficiency can be modulated by its interactions with the substrate protein in areas adjacent to this consensus site^32^, local pH may affect the protonation state of furin recognition elements of these PLTs. We tested this possibility by examining PLT cleavage by human furin under mild reducing conditions in studies performed at pH 7.0 or pH 5.5 (Fig. 4a, b). In comparable conditions, Hmx and Jlx appeared to be more readily cleaved by human furin, with Ahx and Spx the slowest, and PE, Chx, Cfx, and Sfx being intermediate at pH 5.5. As expected, PE was more readily cleaved under acidic but not neutral conditions^30^, a trend that could be observed for most PLTs, with the notable exception of Sfx, which was significantly cleaved by furin at pH 7.0. Remarkably, we could not detect any substantial activation of Ahx in vitro, whereas Hmx and Jlx demonstrated a greater susceptibility to furin. Variation in the level of cleavage despite strong conservation of the consensus furin-recognition motif and the similar accessibility to this site in all PLT structures suggest that other elements might be at play in mediating the furin-toxin interaction. Analysis of the structure and sequence of PLTs hints at a potential role for a loop of domain I, which presents interesting differences in a 3-residue segment in the vicinity of the consensus motif (Figs. 1 and 4c–g). In particular, Hmx and Jlx contain a ‘KDA’ sequence with a unique lysine, whereas the presence of a negatively charged residue at this position in other toxins correlates with weaker furin activation. Remarkably, the corresponding loop in Sfx presents a more neutral ‘GGT’ sequence, that may favour furin cleavage at neutral pH. This secondary site may play a role in stabilizing the activation loop for presentation to furin, or interact directly with the protease. In addition, PE and Chx are known to interact with a number of intermediate receptors that guide them through complex intracellular pathways^24^, it may thus not be excluded that other proteins might mediate the timing and presentation of PMTs to furin for activation.

### Domain III

Following RME and furin processing, vesicular trafficking of PE in non-polarized target cells results in cytoplasmic delivery of the protein’s C-terminal catalytic domain, which is achieved after reaching the ER via retrograde transport, and facilitated by a C-terminal KDEL-like sequence motif^33^. The C-terminal K residue on PE is presumed to be cleaved by a carboxypeptidase to produce the KDEL terminal sequence prior to host cell uptake^34^. A similar process would be inferred for the terminal K present on Chx and Ahx sequences (Fig. 1a). KDEL sequences function in a pH-dependent fashion^35^ and can tolerate alternate amino acids in place of the KD residues^36^, which would be consistent with the terminal KTEL and HDEL sequences for Cfx and Spx, respectively. Hmx, Jlx, and Abx, however, do not terminate with a KDEL, but rather in a T/H-KR sequence, while Sfx ends in a THTN sequence. Interestingly, Abx, which shares 98% amino acid homology with PE, terminates with a HKR sequence and not KDEL(K). These findings are consistent with the suggestion that PLTs may have several potential routes to reaching the ER^37^. In the ER, the furin-cleaved toxin fragment undergoes partial unfolding and is exported to the cytosol by retro-translocation. It has been suggested that PE exploits the endoplasmic-reticulum-associated protein degradation (ERAD) system, which involves the Sec61 translocon^38^, but manages to avoid proteasomal degradation thanks to its low lysine content that averts poly-ubiquitination^39^. Consistent with that observation, there is a paucity of lysine residues in the C-terminal catalytic domain of all PLT family members (Fig. 1a).

**Fig. 5:**
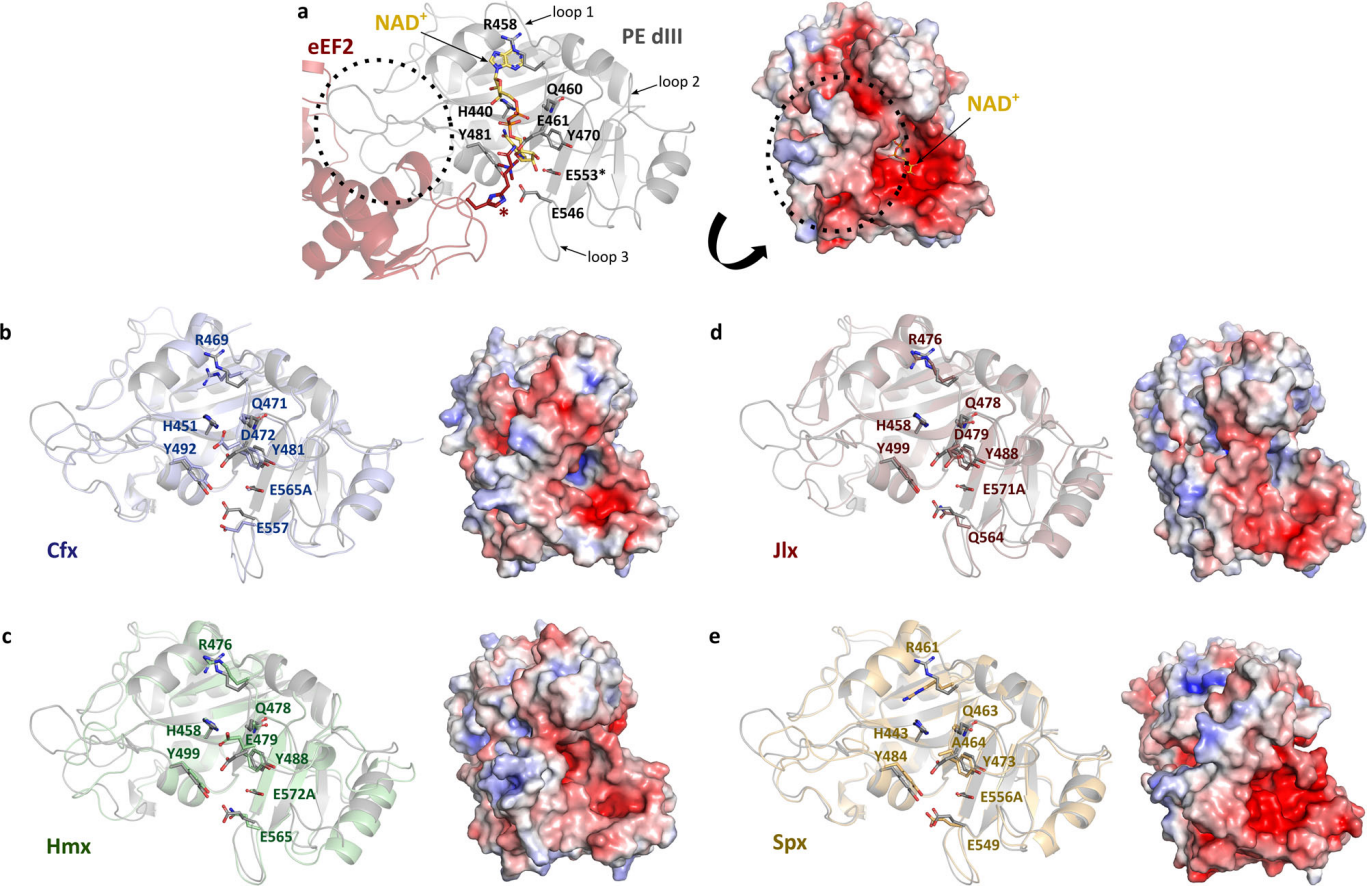
Catalytic domain and ADP-ribosylation activity. **a** Structure of PE domain III (grey) in its complex (PDB 2ZIT) with eEF2 (red) and NAD^+^ (yellow). Residues involved in the catalytic activity and the diphthamide target (red^*^) are shown as sticks, and loops important for activity and substrate recognition are indicated. The arginine-rich eEF2 interaction site in PE is circled, with NAD^+^ included to highlight the position of the catalytic site. **b** *Collimonas* (Cfx; blue), **c** *Chromobacterium* (Hmx; green), **d** *Janthinobacterium* (Jlx; red), and **e** *Shewanella* (Spx; yellow) exotoxins were superposed with PE domain III. Domains I and II were omitted for clarity. Lower panels show the electrostatic surface potential of domain III from each toxin, calculated using the APBS tool in PyMOL (scale from −5, red to +5, blue).

The high potency of PE is due to its ADP-ribosylating activity on cytosolic eukaryotic elongation factor 2 (eEF2), which supports translocation of mRNA and tRNA in the processing of nascent polypeptides on the ribosome. PE specifically causes a single post-translational diphthamide modification of a histidine residue located in domain IV of eEF2^40^. Recognition of eEF2 occurs through a flexible network of electrostatic interactions at the surface of domain III that allows the target diphthamide presentation to the catalytic pocket^6,10^. The eEF2-binding site of PE consists of a positively charged patch involving a series of arginine residues present in several flexible loops (Fig. 5a). Although not fully conserved when comparing primary sequences (Fig. 1a), all PLT structures present similarly located flexible loops that provide a positive surface area that may be compatible with EF2 binding (Fig. 5). The ADP-ribosyltransferase activity of PE involves an active site consisting of a shallow pocket at the surface of domain III composed of the NAD^+^ binding site, and the catalytic site centred around residue E553^5,41^. This glutamic acid forms a hydrogen bond with the ribose moiety of NAD^+^ and positions the dinucleotide for nucleophilic attack by the diphthamide residue, resulting in an ADP-ribosylated eEF2 and a free nicotinamide^10^. In the case of PE, access of the NAD^+^ cofactor to the catalytic pocket is regulated by movement of three surrounding loops (loop 1: 456–462, loop 2: 515–520, loop 3: 546–550) that position amino acids side chains of H440, R458, Q460, E461, Y470, Y481, and E546 in an organization around the E553 residue that is critical for enzyme function (Fig. 5). A similar organization of amino acids in domain III of Chx has been described^41^. In Ahx, the catalytic pocket is composed of the strictly conserved residues E571, H458, Y499, and Y488 and aligns perfectly with the structures of PE and Chx. We superposed the newly identified PLTs with PE and observed extremely high conservation and positioning of residues within the catalytic cleft (Fig. 5), indicating that the NAD^+^ cofactor can be accommodated within this pocket. Remarkably, loops 1 and 2 of all PLTs superpose well with PE, whilst loop 3, which is conserved at the amino acid sequence level, presented more flexibility in our substrate-free structures and may regulate access of the catalytic site to diphthamide. PLTs used for crystallization were enzymatically inactive variants achieved by the replacement of the critical glutamic acid residue with alanine, this mutation did not affect the structure of PE^2^ and it appears that this is also true for these new family members. Although we were not able to produce active toxins due to safety concerns, our structural analysis shows that elements involved in NAD^+^ binding and ADP transferase are conserved in all PLT family members, supporting their putative ADP-ribosylation activity. However, it remains to be confirmed if they may all target eEF2 or disrupt other intracellular processes.

### Evolutionary analysis

**Fig. 6:**
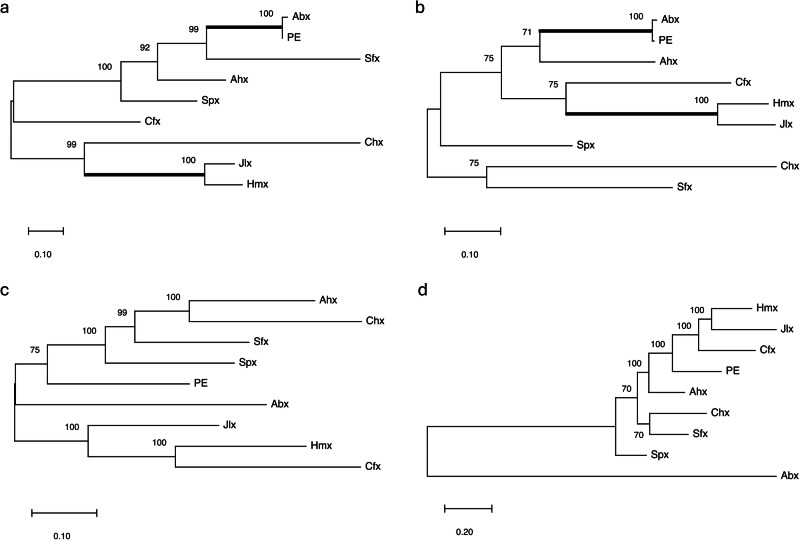
Phylogenetic tree comparison of PLTs. Core protein components analysis. **a** Amino acid sequence relationships for the nine PTLs were inferred using the Maximum Likelihood method and JTT matrix-based model with the highest log likelihood with 713 positions in the final dataset showing the highest log likelihood (−9362.91). **b** DNA sequence relationships for the nine PTLs were inferred using the Maximum Likelihood method and JTT matrix-based model with the highest log likelihood with 2241 positions in the final dataset showing the highest log likelihood (−15,954.258). In both cases, branch lengths represent the number of substitutions per site (potential evolutionary distance) with bootstrap consensus values determined from 1000 replicates shown at each branch. Splits identified in both (**a**) and (**b**) are highlighted (bold line). Comparison of core genome analysis of the nine bacterial genomes for **c** amino acid and **d** DNA sequences. Relationships of the nine PLT amino acid sequences were inferred using the Maximum Likelihood method for 145 core genes collected by BLAST examined for 38857 positions in the final dataset highest log likelihood (−493,105.13) via the JTT matrix-based model. Core genome analysis of the nine bacterial genomes used 145 core genes collected by BLAST examined for 116171 positions in the final dataset highest log likelihood (−917,866.687), being conducted in MEGA11. In both cases, branch lengths represent the number of substitutions per site (potential evolutionary distance), with bootstrap consensus values determined from 1000 replicates shown at each branch.

The newly identified PLTs expand this toxin family to nine members across diverse bacterial species, prompting questions about their origin. To elucidate their evolutionary history, we compared phylogenetic trees from amino acid and DNA sequences of these proteins (Fig. 6a, b). PE and Abx toxins showed close recent relationships in both amino acid and DNA sequences, while Jlx and Hmx also displayed an associated relationship, albeit to a lesser extent. Other PLTs did not show similar organizational patterns, suggesting vertical gene transfer (VGT) to be unlikely. Phylogenetic analyses employ multiple assumptions that could cause artificial differences^42^, therefore, we also performed phylogenetic tree analysis by comparing amino acid and DNA sequences for 145 core genes from each of the bacterial species expressing a PLT, which were aligned individually and recombined into one sequence^43–46^. These trees showed distinct organization between amino acid (Fig. 6c) and DNA (Fig. 6d) sequences and did not suggest any evolutionary relationships between the bacterial species expressing PLTs.

We next examined whether these PLTs could have resulted from foreign gene introduction by comparing toxin sequence guanine–cytosine (GC) content with corresponding core genome sequences^47^. GC content of 145 core genes was used in a probability–probability (PP) test to determine if a normal distribution existed^48^. Except for *Acinetobacter baumannii*, *Aeromonas hydrophila*, and *Janthinobacterium lividum*, genomes closely matched the normal distribution diagonal (Supplementary Fig. 2). A one-sample Kolmogorov-Smirnov test confirmed non-normal GC distribution for these three species. We fitted histograms of non-normal data with normal distribution curves to obtain fitted means and standard deviations for querying potential HGT^49^ (Supplementary Fig. 3). Only PLT genes in *Aeromonas hydrophila*, *Chromobacterium haemolyticum, Collimonas fungivorans*, and *Shewanella putrefaciens* matched the core gene GC content. PLT genes from *Pseudomonas aeruginosa*, *Vibrio cholerae*, *Serratia fonticola*, and *Janthinobacterium lividum* did not, suggesting these genes are not native. DNA analysis therefore indicates that these toxin genes likely entered the bacterial genome long ago through HGT, with subsequent base composition changes occurring over evolutionary time.

To further examine PLT gene transmission, we conducted a base composition analysis of the toxin gene and adjacent areas^50^. We used a sliding window analysis (1000 bp sequence length, 100 bp step) to sample GC content as a criterion for HGT (Supplementary Fig. 4). Most toxin genes showed GC content variations from adjacent sequences, supporting HGT. The GC contents of three toxin genes (PE, Chx, and Cfx) showed ascending trends, while others showed descending trends, not aligning with phylogeny trees for this toxin family (Fig. 6). The close genetic relationship between Abx and PE was not observed here. Overall, the significant GC content variation from adjacent genes, regardless of amino acid homology, supports the potential for HGT.

## Discussion

Here we report the discovery of six previously unknown bacterial toxins that expand the PLT family to nine members. Our analysis of four of these new members by X-ray crystallography presents the defining three-domain structure typical of this family, despite substantial variability in the amino acid sequences of these proteins. Beyond these elements that define the overall fold, all family members have two highly conserved functional components: a furin cleavage site and a catalytic site supporting the ADP-ribosylation activity. These two features provide additional hallmarks for this group of proteins, being essential for their capacity to intoxicate host target cells and function as a potential virulence factor where eukaryotic protein elongation is targeted. PLT family members have now been identified in a wide range of bacterial species, suggesting selective advantage in many different environmental situations. While convergent evolution could explain the existence of one or two of these toxins, a family of at least nine members implies other mechanisms. Our analysis suggests the dissemination of this toxin structure through HGT. It is presumed that subsequent VGT within a species occurred to tune PLT elements, allowing them to acquire unique properties for optimized function within a specific environmental niche.

*Pseudomonas aeruginosa* is a common pulmonary pathogen in cystic fibrosis patients, with PE perceived as an important element in these chronic infections^51^. *Vibrio cholera* is best known for cholera toxin (CT), but non-O1/non-O139 serogroups of this species secrete Chx without the enterotoxicity associated with gastrointestinal infection^52^. *Aeromonas hydrophila*, which can secrete Ahx^4^, is a well-known fish pathogen that more recently has been recognized as an emerging pathogen associated with human gastroenteritis and skin infections^53^. Most of the newly described PLTs have also been associated with soil or aquatic environments, as well as human infections. *Acinetobacter baumannii*, known for its environmental persistence, is one of the most successful pathogens responsible for nosocomial ventilator-associated infections in the modern healthcare system^54^. *Chromobacterium haemolyticum* has been shown to cause human pneumonia following aspiration of river water^55^. *Collimonas fungivorans* has been isolated from fungus^56^ with no reported human infections to date. *Janthinobacterium lividum* is known primarily as a fish pathogen^57^. *Serratia fonticola* has been identified as a pathogen in urosepsis^58^ and biliary tract infection^59^. *Shewanella putrefaciens* can infect fish and cause human respiratory and gastrointestinal infections^60^. Thus, bacterial species capable of expressing PLTs appear to be primarily soil and aquatic in nature, where they could potentially utilize this toxin to infect eukaryotes encountered in those environments.

PE is a virulence factor suggested to help stabilize chronic pulmonary infections of *Pseudomonas aeruginosa* through its capacity to rapidly and efficiently move across polarized epithelial cells, to target and intoxicate cells within the underlying lamina propria^25^. Epithelial transcytosis of PE does not require a catalytically active toxin^25^, and recent studies on Chx have shown that domain I alone is sufficient for transcytosis^22^. Thus, it is not surprising that chimeric proteins containing domain I of some of these PLTs were also capable of A → B transcytosis across the human epithelium of the intestine and/or airway. Transcytosis efficiency of these PLT domain I-containing proteins may vary due to different apical entry receptors and the subsequent intracellular trafficking events resulting from distinctive routes of entry. Indeed, PE has been suggested to enter cells through LRP1^7^ while Chx may use LRP1^3^ and TMEM132A^24^ for receptor-mediated entry. It is unclear if the PLTs identified here could use similar internalization strategies that might allow their function as virulence factors in human infections. Our data shows that the isolated domain I of these proteins can mediate A → B transcytosis across polarized tissues of the human airway and intestine, supporting this possibility.

Once having overcome the epithelial barrier, known PLTs are assumed to rely on the host cells for their activation by furin into a di-chain molecule. This highly conserved furin recognition site was present in all PLTs identified here, although cleavage achieved with human furin occurred with different levels of efficiency. Cytotoxicity associated with this family of toxins is due to their enzymatic activity and the ADP-ribosylation of eEF2. The main structural elements responsible for substrate recognition and catalytic activity in the original PLTs are present and therefore suggest the newly identified proteins hold a similar function. Amino acid sequence variations in sites adjacent to these conserved structures might be important in protein-protein interactions that could optimize furin and/or substrate binding properties to better target specific hosts or host cell types.

Immunotoxins harnessing domains II and III of PE have demonstrated utility in the killing of cancer cells through cytokine^61^ or antibody retargeting^62^. The newly identified PLTs may therefore be useful in extending such therapeutic approaches. Catalytically inactive PLT variants may also be used as mucosal antigen delivery tools, as has been shown with PE^22,25^. Thus, our report provides a framework for the development of anti-toxin therapies and the engineering of novel toxin-based therapeutics using this extended family of bacterial toxins.

## Methods

### Database search with BLAST

A standard local alignment search was performed using the NIH National Library of Medicine online server (https://blast.ncbi.nlm.nih.gov/Blast.cgi). The amino acid sequence of PE (UniProt entry P11439) was used as a template for the BLASTP search against the database of non-redundant protein sequences, with the BLOSUM62 matrix. To filter for known sequences, hits from *Pseudomonas*, *Vibrio*, *Aeromonas* and synthetic constructs were excluded from the start of the search, which was then re-iterated as new organisms were identified.

### Protein expression and purification

For safety considerations, all the exotoxin homologues used in this study correspond to catalytically inactive mutants, by analogy with the previously reported mutations for *Pseudomonas* exotoxin A (E553A) and Cholix (E581A)^3^. Details of DNA and protein sequences are provided in supplementary information. All proteins were codon optimised for *E. coli* expression, synthesised, and cloned into a pET-28a(+) expression vector (GenScript, NJ, USA) with the native signal peptide sequence being replaced with an N-terminal 6xHis-tag and TEV protease recognition site. Expression was carried out in T7 Express Competent *E. coli* (New England Biolabs, Hitchin, UK) cells grown in terrific broth medium at 37 °C for ~3 h and induced with 1 mM final IPTG concentration overnight at 16 °C when cells were harvested and frozen at −80 °C. Cells were lysed by sonication for 15 min on ice, in 0.02 M TRIS pH 8.0 with 0.2 M NaCl and 25 mM imidazole. Proteins were purified by IMAC (immobilized metal ion affinity chromatography) with a HisTrap FF column (Cytiva, Amersham, UK), TEV cleavage of the affinity tag followed by reverse IMAC and size exclusion (Superdex200, Cytiva, Amersham, UK). Final samples were kept in 25 mM TRIS pH 7.5 with 0.15 M NaCl, and 5% glycerol at concentrations between 8–20 mg/mL.

### In vitro transport

Cultures from 3D models of human small intestinal tissues (SMI-100) or airway (AIR-100) epithelial tissues (MatTek Corporation; MA, USA) established on cell culture inserts were allowed to stabilize for 24 h at 37 °C prior to use. Only inserts having a trans-epithelial electric resistance (TEER) of >400 Ω•cm^2^ were considered to have sufficient integrity for use in studies. A secondary verification of monolayer integrity was performed by assessing suppression 70 kD dextran transport. The chambers were washed once with transport buffer (PBS). Test molecules, prepared at a concentration of 20 µg/mL, were applied to the apical surface of inserts in 100 µL volumes. Basolateral volumes of 500 µL PBS were collected at 2 h in AIR-100 and SMI-100 studies for ELISA analysis to determine the extent of transport. The amount of hGH cargo detected in the basal media was used to calculate the apparent permeability (P_APP_) using the following formula:

$${P}_{{{\rm{APP}}}}=\frac{{{\rm{d}}}Q/{{\rm{d}}}t}{A\times C0}$$

Where d*Q* is the change in the amount of hGH in the basal chamber (µg), d*t* is the change in time (s), *A* is the diffusion area (cm^2^), and *C*_*0*_ is the initial concentration of hGH in the apical chamber (µg/mL). Assessment of background leak was performed with hGH alone. Each experimental condition was performed in triplicate.

### ELISA

The extent of PLT-hGH chimaeras transport across SMI-100 or AIR-100 monolayers was determined using an ELISA format where captured PLT-hGH chimaeras were detected using an anti-hGH ELISA kit (Quantikine DGH00; R&D Systems, MN, USA) to quantitate hGH. Absorbance values for the enzymatic reactions at 405 nm were registered in an ELISA microplate reader (Bio-Rad, CA, USA).

### Furin proteolytic activation assay

For activation of PLTs by furin, all proteins (25 µg) were incubated with or without 2 units of recombinant human furin (#P8077L, NEB, UK) for 1 hr at 25 °C in either 20 mM MES, 0.1% Triton X-100, 0.2 mM CaCl2, 0.2 mM 2-Mercaptoethanol (pH 5.5) or 20 mM HEPES pH 7.0, 0.1% Triton X-100, 0.2 mM CaCl2, 0.2 mM 2-Mercaptoethanol (pH 7). Reactions were stopped by the addition of NuPAGE LDS sample buffer (Invitrogen, UK), and 1 µg of protein was loaded onto an SDS-PAGE gel in the reduced buffer.

### X-ray crystallography

Original crystallisation conditions for each protein were identified by screening using an automated platform (Crystal Phoenix, Art Robbins Instruments, CA, USA), using a sitting drop set-up. Crystals of Hmx were grown within 3–4 days with 0.1 μL of the sample at 8 mg/mL mixed with 0.1 μL of reservoir solution consisting of 0.12 M monosaccharides, 0.1 M buffer system 1 pH 6.5, 37.5% v/v precipitant mix 4 (condition F4 of the Morpheus Screen, Molecular Dimensions, UK); Crystals of Jlx were grown within 8-10 days with 0.1 μL of the sample at 18 mg/mLl mixed with 0.1 μL of reservoir solution consisting in 0.1 M amino acids, 0.1 M buffer system 1 pH 6.5, 30% v/v precipitant mix 1 (condition H1 of the Morpheus Screen, Molecular Dimensions, UK). Crystals of Spx were grown within 2-3 months with 0.2 μL of the sample at 20 mg/mL mixed with 0.1 μL of reservoir solution consisting of 0.2 M magnesium chloride hexahydrate, 0.1 M HEPES pH 7.0, 20% w/v PEG 6000 (condition C10 of the PACT premier Screen, Molecular Dimensions, UK); Crystals of Cfx were grown within 5–7 days with 0.2 μL of the sample at 19 mg/mL mixed with 0.1 μL of reservoir solution consisting in 0.2 M magnesium chloride hexahydrate 0.1 M BIS-Tris pH 5.5, 25% w/v PEG 3350 (condition H11 of the JCSG-plus Screen, Molecular Dimensions, UK). Crystals of Spx and Cfx were transferred briefly into a cryo-protectant solution, consisting of the growth condition supplemented with 10% glycerol, before freezing in liquid nitrogen. Diffraction data were collected at stations I03 and I04 of the Diamond Light Source (UK), equipped with an Eiger2 XE 16 M detector (Dectris, Switzerland). Complete datasets were collected from single crystals at 100 K. Raw data images were processed and scaled with DIALS^63^ and AIMLESS^64^ using the CCP4 suite 7.0^65^, or STARANISO for Jlx^66^. Molecular replacement was performed with the coordinates of the individual domains from PE (PDB code 1IKQ^67^) or Cholix (PDB code 2Q5T^3^) to determine the initial phases for structure solution in PHASER^68^. Working models were refined using REFMAC5^69^ and manually adjusted with COOT^70^. Water molecules were added at positions where Fo−Fc electron density peaks exceeded 3σ and potential hydrogen bonds could be made. Validation was performed with MOLPROBITY^71^. Crystallographic data statistics are summarised in Table 1. The atomic coordinates and structure factors (PDB codes 9G7M, 9G7N, 9G7O, and 9G7P) have been deposited in the Protein Data Bank (http://wwpdb.org). Figures were drawn with PyMOL (Schrödinger, LLC, NY, USA).

### Evolutionary analysis

Phylogenetic trees were constructed in MEGA11^72^ using the Jones–Taylor–Thornton (JTT) matrix-based model and the Maximum Likelihood (ML) method. A heuristic approach was applied by automatically applying the Neighbour-Join (NJ) and BioNJ algorithms to a matrix of pairwise distances computed by the JTT model, and then selecting the topology with the highest log likelihood value. Guanine–Cytosine (GC) base pairs from the nucleotide sequences of the toxin genes and their surroundings were analysed using custom-written scripts in Python. The window was intended to be 1000 bp long from which the sequence was retrieved, and samples were regularly taken at intervals of 100 bp. The sampling region spanned a range of 14,000 bp, that is, 6000 bp away from either terminal of the toxin gene fragment. A smooth curve made up of random points was plotted with the GC contents of the DNA sequences.

### Statistics and reproducibility

Statistical analyses were performed using GraphPad Prism software version 10.0. Results are presented as mean ± SEM of a representative experiment. The sample size is indicated in the figure legend. A one-way ANOVA test was used and showed there was significant variance between the data sets, with ^*^p < 0.05 compared to the control group in a Bonferroni post-test.

### Reporting summary

Further information on research design is available in the Nature Portfolio Reporting Summary linked to this article.

## Supplementary information

Supplementary Information

Description of Additional Supplementary Files

Supplementary Data 1

Reporting summary

## Data Availability

The atomic coordinates and structure factors (PDB codes 9G7M, 9G7N, 9G7O, and 9G7P) have been deposited in the Protein Data Bank (http://wwpdb.org). Data sets generated during the current study are available from the corresponding author upon reasonable request. Original data for Fig. 3f, g are provided in Supplementary Data 1, and pictures for Fig. 4 are available in Supplementary Fig. 5.

## Code availability

The underlying code for evolutionary analysis in this study is available in [PLTs_GC] and can be accessed via this link [https://github.com/YYWANG-code/PLTs_GC.git]. 10.6084/m9.figshare.28279925.v1
