# Supplementary material for: Discovery of mono-ADP ribosylating toxins with high structural homology to Pseudomonas exotoxin A
Source: Commun Biol. 2025 Mar 11;8:413. doi: 10.1038/s42003-025-07845-y (PMC11897225; doi:10.1038/s42003-025-07845-y)
Supplement: Supplementary file 1 — Supplementary Information [file 42003_2025_7845_MOESM1_ESM.pdf]

Supplementary information

Supplementary Table 1. Pseudomonas-like toxins (PLTs) examined in these studies.

Bacterial species	Accession#	Abbr.	%GC	MW (Da)	pI
Pseudomonas aeruginosa	AKG00567.1	PE	68	66,755	5.28
Vibrio cholera	QKU75906.1	Chx	43	70,704	5.12
Aeromonas hydrophila	APJ14853.1	Ahx	64	66,596	5.15
Acinetobacter baumannii	SCY08530.1	Abx	66	68,969	5.36
Collimonas fungivorans	PFH04378.1	Cfx	58	71,413	6.77
Chromobacterium haemolyticum	OQS34021.1	Hmx	62	68,085	6.05
Janthinobacterium lividum	AMC33769.1	Jlx	57	73,057	6.01
Serratia fonticola	QIP94533.1	Sfx	45	69,451	5.17
Shewanella putrefaciens	WP_152829160.1	Spx	49	71,042	5.00

Accession# identifiers were obtained from GenBank. Abbr. = three-letter abbreviation used to define each PLT. %GC = Relative guanine-cytosine content. MW (molecular weight in Daltons) and pI (isoelectric point) were calculated using https://web.expasy.org/compute_pi/.

Supplementary Figure 1. Comparison of amino acid homology of Pseudomonas-like toxins (PLTs) for a) the overall protein, b) domain I, c) domain II, and d) domain III. Toxin amino acid sequences were aligned in pairs with identities of each pair obtained and gathered using DNAMAN. Mean homology scores for overall protein, domain I, domain II, and domain III were 47.9%, 42.7%, 47.4%, and 54.9%, respectively.

Overall Homology

	PE	Chx	Ahx	Abx	Cfx	Hmx	Jlx	Sfx	Spx
PE	100.00	33.22	63.88	98.14	48.44	39.12	39.25	58.39	58.29
Chx	33.22	100.00	34.67	32.19	37.85	38.08	38.04	32.55	35.29
Ahx	63.88	34.67	100.00	63.73	50.65	41.41	42.06	52.30	63.22
Abx	98.14	32.19	63.73	100.00	47.29	38.30	38.78	57.63	57.67
Cfx	48.44	37.85	50.65	47.29	100.00	48.59	49.25	42.48	49.75
Hmx	39.12	38.08	41.41	38.30	48.59	100.00	84.39	37.41	41.62
Jlx	39.25	38.04	42.06	38.78	49.25	84.39	100.00	38.23	43.46
Sfx	58.39	32.55	52.30	57.63	42.48	37.41	38.23	100.00	50.33
Spx	58.29	35.29	63.22	57.67	49.75	41.62	43.46	50.33	100.00

Domain I Homology

	PE	Chx	Ahx	Abx	Cfx	Hmx	Jlx	Sfx	Spx
PE	100.00	28.28	59.04	100.00	40.73	33.74	33.20	47.58	51.60
Chx	28.28	100.00	32.11	27.82	35.20	37.07	36.54	26.45	33.06
Ahx	59.04	32.11	100.00	58.57	43.60	36.73	35.77	43.09	60.56
Abx	100.00	27.82	58.57	100.00	40.08	33.20	32.66	47.01	51.18
Cfx	40.73	35.20	43.60	40.08	100.00	41.6	41.43	35.37	42.06
Hmx	33.74	37.07	36.73	33.20	41.60	100.00	91.15	32.78	35.63
Jlx	33.20	36.54	35.77	32.66	41.43	91.15	100.00	33.06	35.89
Sfx	47.58	26.45	43.09	47.01	35.37	32.78	33.06	100.00	42.74
Spx	51.60	33.06	60.56	51.18	42.06	35.63	35.89	42.74	100.00

Domain II Homology

	PE	Chx	Ahx	Abx	Cfx	Hmx	Jlx	Sfx	Spx
PE	100.00	30.99	60.84	98.61	40.14	40.14	40.14	62.94	57.34
Chx	30.99	100.00	33.11	30.07	36.30	39.46	37.41	34.27	32.87
Ahx	60.84	33.11	100.00	60.42	43.24	42.95	43.62	53.47	60.42
Abx	98.61	30.07	60.42	100.00	39.86	39.86	39.86	61.81	56.94
Cfx	40.14	36.30	43.24	39.86	100.00	55.33	56.67	36.36	46.15
Hmx	40.14	39.46	42.95	39.86	55.33	100.00	86.84	36.36	43.36
Jlx	40.14	37.41	43.62	39.86	56.67	86.84	100.00	35.66	45.45
Sfx	62.94	34.27	53.47	61.81	36.36	36.36	35.66	100.00	48.61
Spx	57.34	32.87	60.42	56.94	46.15	43.36	45.45	48.61	100.00

Domain III Homology

	PE	Chx	Ahx	Abx	Cfx	Hmx	Jlx	Sfx	Spx
PE	100.00	40.87	71.96	95.38	62.67	45.50	46.70	69.01	66.82
Chx	40.87	100.00	39.71	39.47	42.51	38.27	40.41	38.54	39.22
Ahx	71.96	39.71	100.00	72.31	64.02	46.00	48.22	63.38	68.22
Abx	95.38	39.47	72.31	100.00	62.05	44.09	46.45	68.21	66.67
Cfx	62.67	42.51	64.02	62.05	100.00	52.50	53.81	55.87	62.15
Hmx	45.50	38.27	46.00	44.09	52.50	100.00	73.89	43.50	48.00
Jlx	46.70	40.41	48.22	46.45	53.81	73.89	100.00	46.70	51.27
Sfx	69.01	38.54	63.38	68.21	55.87	43.50	46.70	100.00	61.03
Spx	66.82	39.22	68.22	66.67	62.15	48.00	51.27	61.03	100.00

100.00 20.00

Supplementary Table 2. PLTs sequence and structure alignments performed with Clustal and TM-align with comparison to *Pseudomonas aeruginosa* exotoxin A (PE) and Cholix (Chx), for the full-length toxin (**a**) and domain I (**b**).

a

	PE		Cholix	
	% ID	rmsd (Å) [Cα][1]	% ID	rmsd (Å) [Cα][1]
PE	-	-	33.2	3.2 [561]
Chx	33.2	3.2 [561]	-	-
Ahx	63.9	2.5 [593]	34.7	2.9 [575]
Hmx	39.1	2.7 [581]	38.1	2.9 [583]
Jlx	39.3	2.7 [577]	38.0	2.4 [579]
ShE	58.3	2.3 [593]	35.3	2.8 [571]
Cfx	48.4	2.6 [589]	37.9	2.9 [572]

b

	PE		Cholix	
	% ID	rmsd (Å) [Cα][1]	% ID	rmsd (Å) [Cα][1]
PE	-	-	28.3	2.6 [219]
Chx	28.3	2.6 [219]	-	-
Ahx	59.0	2.0 [240]	32.1	2.3 [225]
Hmx	33.7	2.4 [238]	37.1	2.0 [237]
Jlx	33.2	2.5 [238]	36.5	2.0 [237]
ShE	51.6	2.0 [238]	33.1	2.3 [224]
Cfx	40.7	2.1 [236]	35.2	2.0 [223]

[1] Number of aligned Cα atoms.

Supplementary Figure 2. P-P (probability–probability) plots of guanine-cytosine (GC) content in 145 core genes shown individually for (**a**) *Acinetobacter baumannii,* (**b**) *Pseudomonas aeruginosa,* (**c**) *Aeromonas hydrophila,* (**d**) *Vibrio cholerae,* (**e**) *Chromobacterium haemolyticum,* (**f**) *Collimonas fungivorans* , (**g**) *Serratia fonticola,* (**h**) *Shewanella putrafaciens,* and (**i**) *Janthinobacterium lividum*. Diagonal lines describe the normal distribution when GC expected and observed GC content are equivalent.

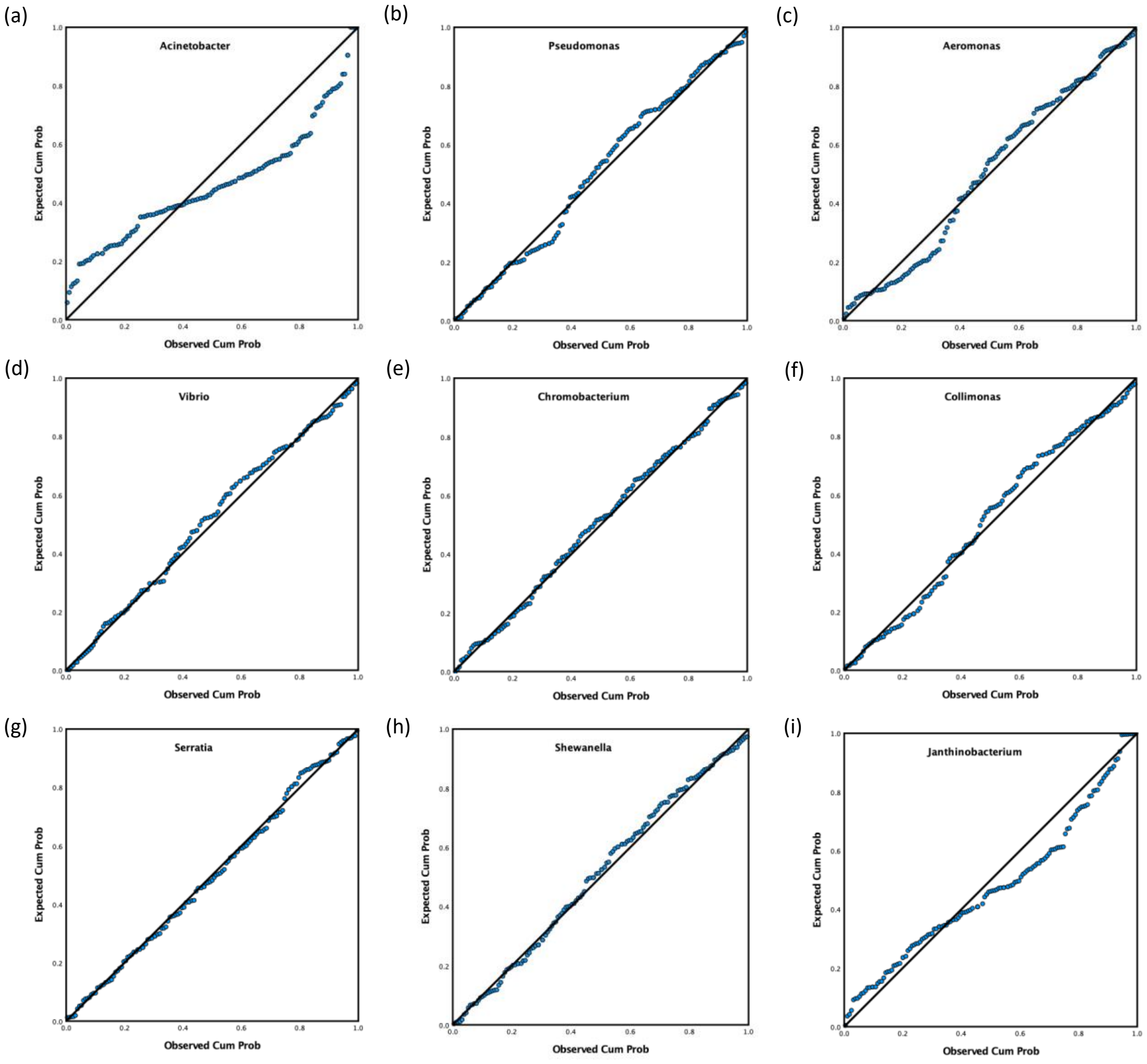

Supplementary Figure 3. Comparison of toxin gene and core gene guanine-cytosine (GC) content. GC content observed in 145 core genes plotted as histograms with fitted normal distribution trendlines and where the straight line represents CG content for the corresponding PLT (**a**) *Acinetobacter baumannii,* (**b**) *Pseudomonas aeruginosa,* (**c**) *Aeromonas hydrophila,* (**d**) *Vibrio cholerae,* (**e**) *Chromobacterium haemolyticum,* (**f**) *Collimonas fungivorans* , (**g**) *Serratia fonticola,* (**h**) *Shewanella putrafaciens,* and (**i**) *Janthinobacterium lividum*.

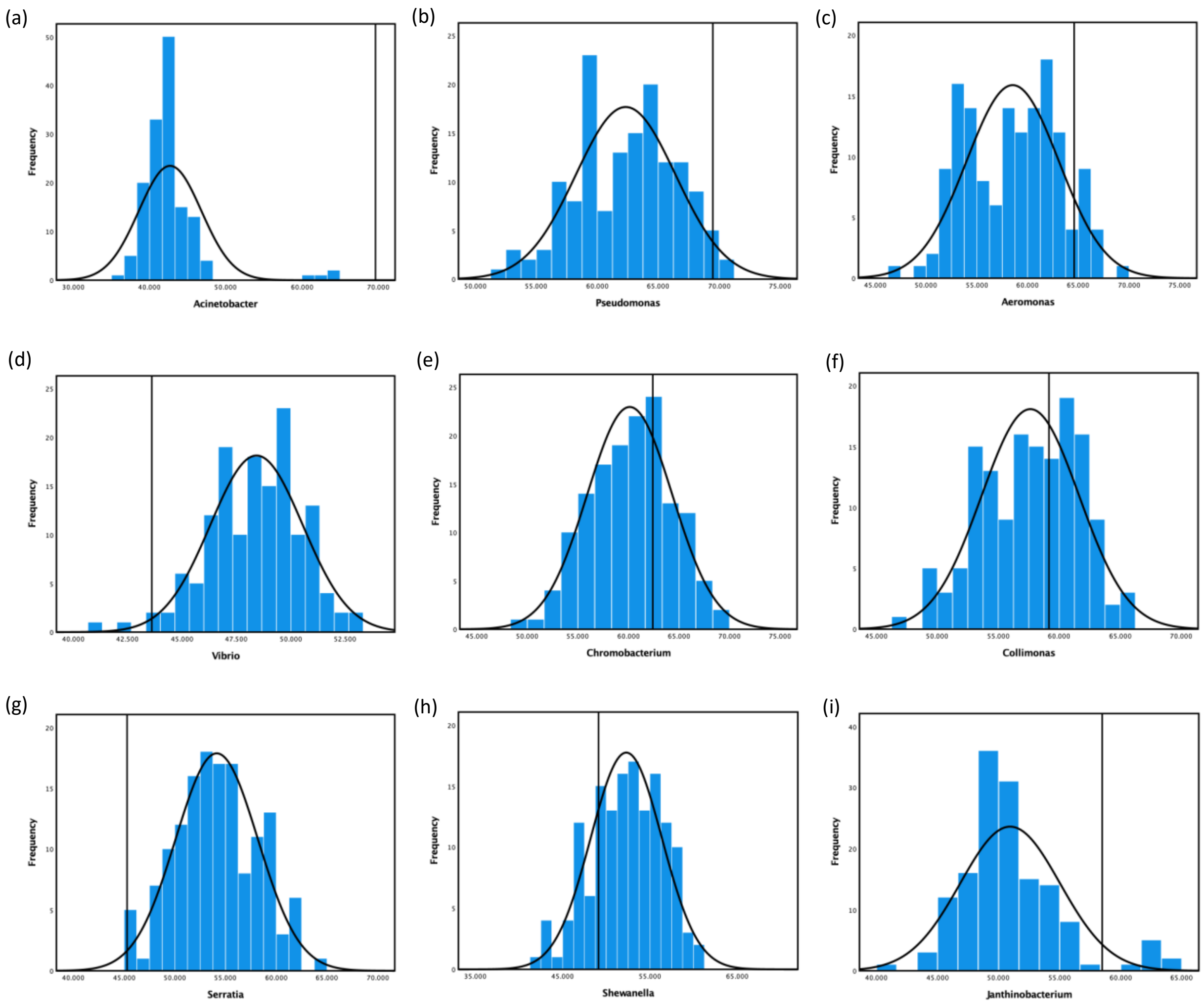

Supplementary Figure 4. Base composition analysis of toxin gene neighbourhood. It was a sliding window analysis that included calculation of GC content of toxin gene and the regions around (4400 – 6000 bp away from either side). The length of every tested sequence is 1000 bp and the interval between the start positions of every two consecutive sequences is 100 bp. (a-i) It was clearly shown separately that the trends of GC contents of mARTs gene in *Acinetobacter*, *Pseudomonas*, *Aeromonas*, *Vibrio*, *Chromobacterium*, *Collimonas*, *Serratia*, *Shewanella*, and Janthinobacterium. The data from toxin gene were highlighted in orange and those from other coding genes within the tested region were marked in green.

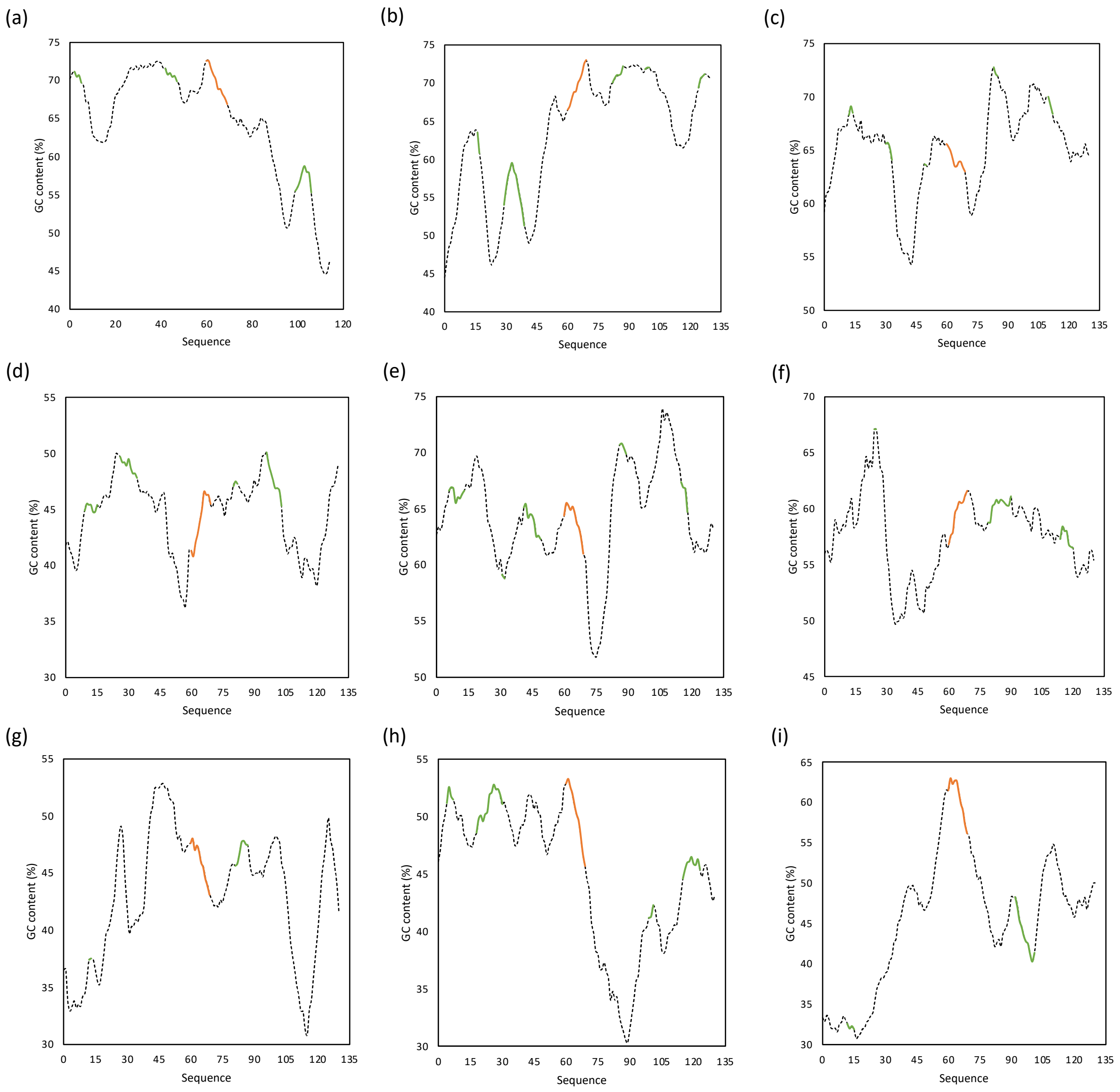

Supplementary Figure5. Original SDS PAGE pictures from the furin cleavage assay (Figure 4)

(a) Furin cleavage of PLTs at pH 5.5 (#1):

MW Marker, *Shewanella, Janthinobacterium*, *Chromobacterium, Collimonas and Vibrio* exotoxins (without or with furin, respectively)

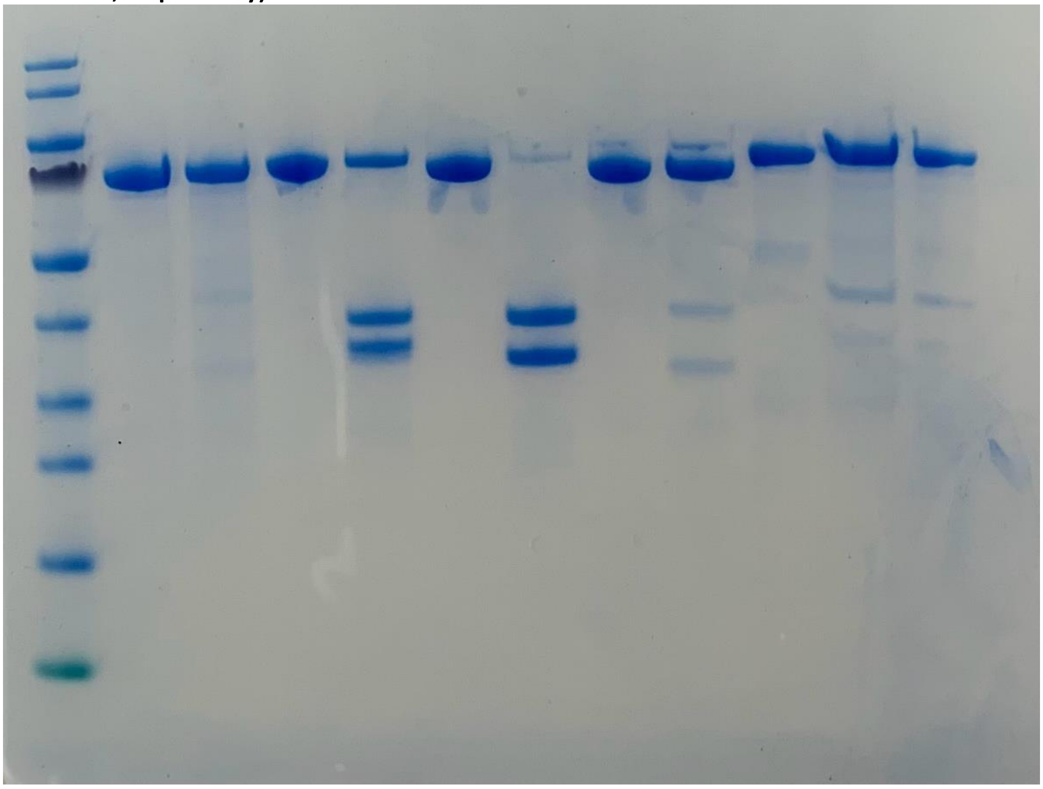

(b) Furin cleavage of PLTs at pH 5.5 (#2):

MW Marker, *Pseudomonas, Aeromonas*, *and Serratia* exotoxins (without or with furin, respectively)

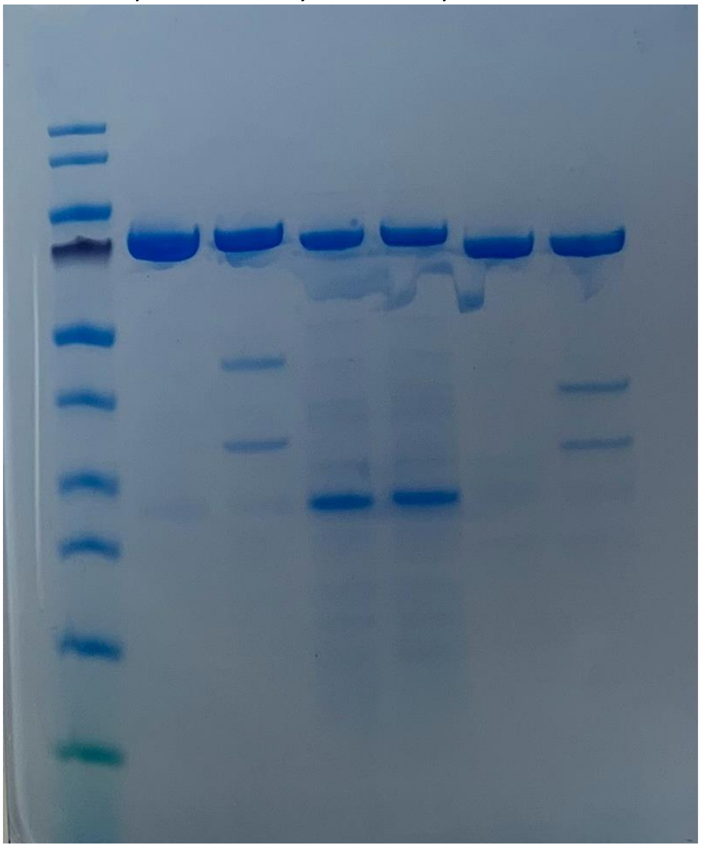

(c) Furin cleavage of PLTs at pH 7.0 (#1):
MW Marker, *Shewanella, Janthinobacterium*, *Chromobacterium, Collimonas and Vibrio* exotoxins (without or with furin, respectively)

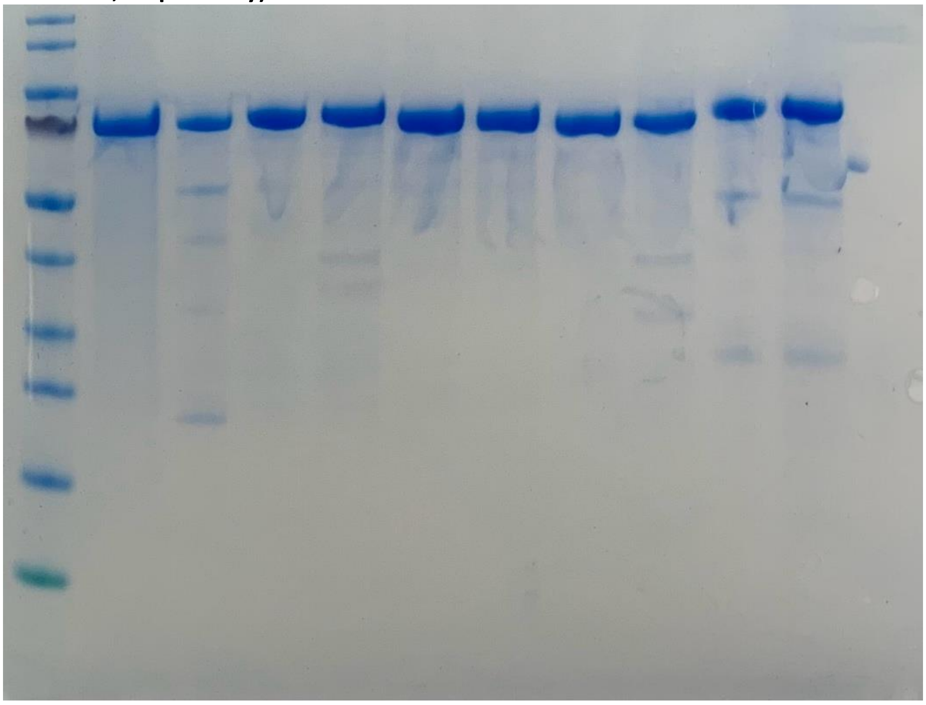

(d) Furin cleavage of PLTs at pH 7.0 (#2):
MW Marker, *Pseudomonas, Aeromonas*, *and Serratia* exotoxins (without or with furin, respectively)

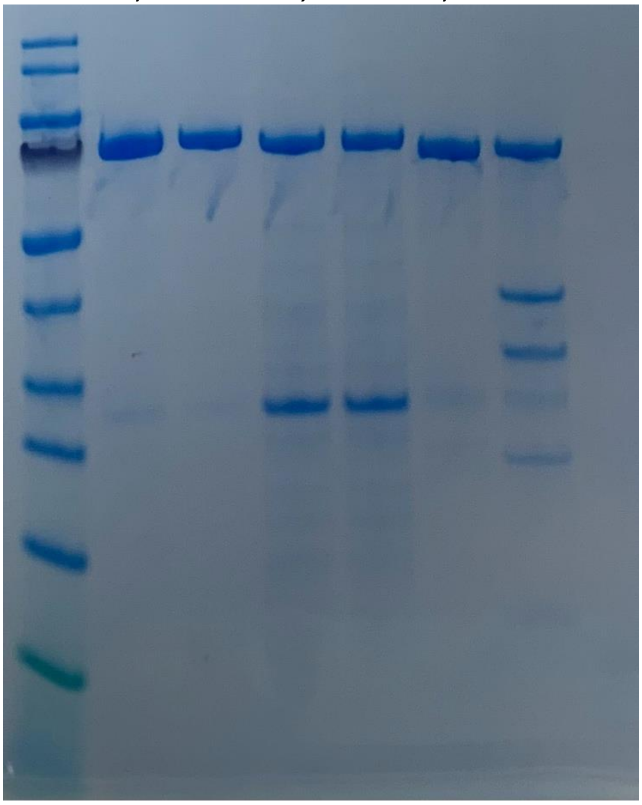

Protein sequences from database. The predicted signal peptide is highlighted in grey.

> *Pseudomonas aeruginosa* exotoxin A (PE)

MHLTPHWIPLVASLGLLAGGSFASAAEEAFDLWNECAKACVLDLKDGVRSSRMSVDPAIADTNGQGVLHYSMVLEGGNDALKLAIDNALSITSDGLTIRLEGGVEPNKPVRYSYTRQARGSWSLNWLVPIGHEKPSNIKVFIHELNAGNQLSHMSPIYTIEMGDELLAKLARDATFFVRAHESNEMQPTLAISHAGVSVVMAQAQPRREKRWSEWASGKVLCLLDPLDGVYNYLAQQRCNLDDTWEGKIYRVLAGNPAKHDLDIKPTVISHRLHFPEGGSLAALTAHQACHLPLETFTRHRQPRGWEQLEQCGYPVQRLVALYLAARLSWNQVDQVIRNALASPGSGGDLGEAIREQPEQARLALTLAAAESERFVRQGTGNDEAGAASADVVSLTCPVAAGECAGPADSGDALLERNYPTGAEFLGDGGDISFSTRGTQNWTVERLLQAHRQLEERGYVFVGYHGTFLEAAQSIVFGGVRARSQDLDAIWRGFYIAGDPALAYGYAQDQEPDARGRIRNGALLRVYVPRSSLPGFYRTGLTLAAPEAAGEVERLIGHPLPLRLDAITGPEEEGGRLETILGWPLAERTVVIPSAIPTDPRNVGGDLDPSSIPDKEQAISALPDYASQPGKPPREDLK

> *Vibrio cholerae* Cholix (Chx)

MYLTFYLEKVMKKMLLIAGATVISSMAHPTFAVEDELNIFDECRSPCSLTPEPGKPIQSKLSIPSDVVLDEGVLYYSMTINDEQNDIKDEDKGESIITIGEFATVRATRHYVNQDAPFGVIHLDITTENGTKTYSYNRKEGEFAINWLVPIGEDSPASIKISVDELDQQRNIIEVPKLYSIDLDNQTLEQWKTQGNVSFSVTRPEHNIAISWPSVSYKAAQKEGSRHKRWAHWHTGLALCWLVPMDAIYNYITQQNCTLGDNWFGGSYETVAGTPKVITVKQGIEQKPVEQRIHFSKGNAMSALAAHRVCGVPLETLARSRKPRDLTDDLSCAYQAQNIVSLFVATRILFSHLDSVFTLNLDEQEPEVAERLSDLRRINENNPGMVTQVLTVARQIYNDYVTHHPGLTPEQTSAGAQAADILSLFCPDADKSCVASNNDQANINIESRSGRSYLPENRAVITPQGVTNWTYQELEATHQALTREGYVFVGYHGTNHVAAQTIVNRIAPVPRGNNTENEEKWGGLYVATHAEVAHGYARIKEGTGEYGLPTRAERDARGVMLRVYIPRASLERFYRTNTPLENAEEHITQVIGHSLPLRNEAFTGPESAGGEDETVIGWDMAIHAVAIPSTIPGNAYEELAIDEEAVAKEQSISTKPPYKERKDELK

> *Aeromonas hydrophila* exotoxin A (Ahx)

MAMLGGAISPVMADESFNLWQECATRCTLDLAQGVRASQLDVASLLGGQTDSGVLHYSMVLEEGGDSLKLALGNALTLRTDGTTITLTSATAGKGPRTYSYTRQGRGNWSLHWLVPVGDDAPASIKVFFHELDAGSEVSHISPIYSIEVSDDLLRTMASNSTLFVRHVENNEINRSLTLSAAGVGFVAAPTQHSRQKRWSEWHTGKVLCLLDPLDAVYNYLSQRTCNLGDTWEGKVYRVLAGAPASHDTHIVPTAISHRLHFAKGDGLAALTTHQVCAIPLESLARSRQPRGWEELSQCGYPVHNLVTLYLLTRLPWSQLDTVITQALANTTPEDGSTPRGQLAQAIRENPAQARLALSMAAAQSDAFSHQQAGNSQEQAASADVVNLTCPAADLNCLAPADSADALQERDYPNGDSFLGDGDEVSFSTAGTRNWSVTRLEQVHRQLLARGYLFVGYHGTFLEAAHSIVFEGVHERDQSSIAPWQGFYVAGDPALAYGYAQDQEADARGRIRNGVLLRVYVPRAALPRLYATQQTLADPGAVDEVGRLIGHPLPLQLEAITGPEEEGGRLETILGWRLAEQAVVIPSTIPTDPRNVGGNLDPASVPQEESAISSLPDYTTQPREDLK

> *Chromobacterium haemolyticum* exotoxin A (Hmx)

MRLYKSLALAAGLIPLLSACETDEASPFAAGTAQTASFKQAASSVIKTDFEIFKQCADNCILSPAEPGKFISTSLPLQITPSPDEGVLYYSMFVQDRFAAAANNSATIKIDEFAKVRINDGQGTGHAPGTLTIELATPDGKVKKFTHKRRTEWFTLNWVVPIGKDAPTSIKLFIMDMDSNKKIVDHSPLYSVDLDDAALARWPDKAKLAFSSANPRNDIILSWPGVGYTAAPTQHNRQKRWSEWHSGILLCWLDPLDAIYNYVTQNRCQLNKTWEGKLYQVVAGKPQINEFKPLAKAPIQHRVHFSKENALGALSAHRVCGIPLESLARSRQPRGWEELSACGYRVESIVGLYIATRLSFDRFRQVVDDLIHSRPVSGAQDPEALEQLGTAVRETPGLAREGLAEAEALLDTYLDYHPGASADDAQRADVLSLTCPADSEPCAAANADGAHVNLEYHPGSSFFAPGELVEFLSNGTTSNWSQERLLATHQRLLDQGYVFAGYHGGSTIAARSIVTGGITPRTQELPPIWKGFYIAGNPEVAYGYALDNDNPRSRGIMMRIYVPRTALPQLFRTSQPLSDEAAALREMSRLFGRNVTLDSTLGYESITGPQAPGEADETVLGWLMARHSVAIPSMIQGNGNNAGKIDVPDYEKKISALPDYVTKR

> *Collimonas fungivorans* exotoxin A (Cfx)

MSKFMSQRWIYLFGWLFLLGTGSTRANTIEGNFRLWDDCSSYCLVYAAPHKIYQTPLATQAVPDSPGKEGVLHYSMVMKDYVGNGQVLALRLDDFATVFVEQESLRLSLLGSDGKPRNFQYARQGAKHWSLNWLVPVGDDAPTSIKVFFKNLDGQNNILSISPLYSVEVDDKTLARWPALATFSVTQENVTQGQGLLGIRRAGVSYVAAPVNHDRHKRWSEWHSGKLLCLLDPLDAIYNYVSQNRCSLGETWEGAIYQTLAGRPVDKYAPPASKPVISQRIHFAKGNALEALTSHRVCGIPLESLARRRKPRGWEEWSSCGNPAANFVALYIATRLPFDQFR

QVIHNLVHGQAVAAPDPVPLDALRTAVIEQPELARQSIAQAADIFRNYQAANPGASAAAAQQADVLAVTCPADARPCGSGASSGVLVQRENPTGAHFLNDGELPSFTVQGTQNWNLNRLQAAHLRLQVQGYVFAGYHGTSLEGAQSIVFGGIHNRQQDLEEIWRGLYVAGDPALAYGYAQDAEGDERGRIRNGTMLRVYVPRSALPRLFATSLPLDHPGASQEVARLIGHPLPLLYESITGPEAAGGNRLETILGWQLAEQAVAIPSMIPTNSRTVGNPLDPATVTLEEKQISSLPGYATKPAKDDKTEL

> *Shewanella putrefaciens* exotoxin A (Spx)

MIKTRYLCLSVAISSLFLAITSVNATDLRGLEPQITSAAQVNEAFDLWQECATHCQLDLSQGIRSSELDLTPLFETSNEEGILHYSMLLGEGNEGLKLAIDNALTLHTTHSTINFTSETAESGPRSYSYIRKGENNWSLNWLVPVGDDAPASIKIFFLEQDAVGLNRYISPIYSIEVSNNLLNSLAHKSTFYIRAFDNNQTLSMVNISSAGVSYVAAPQQHHRQKRWSEWHTGKLLCFLDPFDAFYNYVTQHTCNPDDTWEGQIYRVLAGNPATLDTTAPSTTPAVISHRIHFDRGNSLASLTAHQVCGIPLESLARTRHPRGWEELNNCGYPVRNLVSLFILARLSWDRVEQVIHNALTNPTPGNALDDAIREAPERARVTLTLAAAQVNQFDNQAAGNTPEQAQSADVVSLSCSAGALHCSAPADSANALLEREHPNGANFLGAGEAVSFTTRGTRNWSSARLNHAHQQLIARGYVFVGYHGSSLEGAQSIVFGGIRTRTQALDDVWQGLYISGDPAVAYGYAQDQEPDSRGRIRNGTMLRVYVPGTATAYLYETPLTLADPEAVDAVGHLIGHPLPLQTEAITGPEEAGGRPETILGWELAEQAVAIPSTIPTDPSNIGGDLDPSSIPDEESDISALPDNVTKPHHDEL

> *Janthinobacterium lividum* exotoxin A (Jlx)

MMRLYKGFAIAAGLIPLLSGCVTEEVRPSATEEVRPSNTVAAAGFQQAASSIIKTDFEIFKQCADSCILSPSEPGKFISTSLPVQITPSPDEGVLYYSMFVQDRFASAANNSATIKIDDFAKIRINDGYDTGSAPGSLTIELATPDGQVKKFTHKRRTEWFTLNWAVPIGKDAPTSIKLFIMDIDSNKKIVDHSPLYSVDLDDAALARWPAKAKLAFSSANAMNDIILSWPGVGYTAAPTQHSRQKRWSEWHSGILLCWLDPLDAIYNYVTQNRCQLKNTWEGSLYKVVAGNPQINEFKPVAKAPIQHRVHFSKENALGALSAHRVCGIPLESLARSRQPRGWEELSACGFRVENIVGLYIATRLSFDRFRQVVEDLIHSRPVSGAQDPVALEQLGSVVRETPELAREGLAEAEARLNAYRANHPGSSADDAQRADVLSLTCPADSAPCNAPDAAGAHVNREYHPGTDYFAPGEPVEFLPNGTTRNWSQDRLLATHQRLLDLGYVFAGYHGGSIAAARSIVTGGITRRSQDLQPIWRGLYIASDPEVAYGYALDNNTGATGTMMRVYVPGTALSRLFRTNQALSDEAAALREIGRLLGRNITLADTLGYESITGPQDPGDPDETILGWDLAEHAVVIPSMIRGNNGSQLNVPDYEKKISPLPNYVTKR

> *Serratia fonticola* exotoxin A (Sfx)

MSNKAKLRTAAIAIILASYLSLPAFANDKFDLWTQCADSCSLDLSNQRIWSSPMDLSLFGQTSEQGVLHFSMVLGENNDSVRIGIDNAFSLLINENSIQFKGVSDITKPVKFNYTRQDKGNISVNWLVPIGGGTPSEIKVFIHEVNSANQITSMSPIYTIPVSNEVLSTLEKKSTIYIRGTNSNSLSYELIVKNAGVSIASTQSGPTRKRRWTHWDSGKTLCFIDPLNAIYNYLSQNTCQLDDTWEGKVYLTLYGSPATHDIEMPASPISTRTHFSEGGSLAALTAHRVCAIPFETFTRHRQPRGWEDLEKCGYPVHSLIAFYIAARHSWSQVDQVIQNALDNQESDSDLDRAIRESPQQSRLALTTAAQTSAVFVSQSSNNTEASAANADILTLTCPLSGLNCMVPADGNEAHQERIYPTGAIFLGDGDDISFTTRGTQNWTTDRLIAAHQQLTSQGYVFVGYHGTFLEAAHSIVFEGVRARSQSLDNAWHGFYIAGDPAVSYGYALDQDPDPNTGRIRNGVMLRVYVRQESLPNFYQTNVALSSPDAVNAVSSLIGHTLPLQLDSITGQEDEEGRLETIIGWPLAEQLVVIPSTISTDPRNPGGVLDPSSIPEAEKAISVLPNYSTSPTNTH

> *Acinetobacter baumannii* exotoxin A (Abx)

MHLTPHWIPLVASLGLLAGGLSASAAEEAFDLWNECAKACVLDLKDGVRSSRMSVDPAIADTNGQGVLHYSMVLEGGNDALKLAIDNALSITSDGLTIRLEGGVEPNKPVRYSYTRQARGSWSLNWLVPIGHEKPSNIKVFIHELNAGNQLSHMSPIYTIEMGDELLAKLARDATFFVRAHESNEMQPTLAISHAGVSVVMAQAQPRREKRWSEWASGKVLCLLDPLDGVYNYLAQQRCNLDDTWEGKIYRVLAGNPAKHDLDIKPTVISHRLHFPEGGSLAALTAHQACHLPLETFTRHRQPRGWEQLEQCGYPVQRLVALYLAARLSWNQVDQVIRNALASPGSGGDLGEAIREQPEQARLALTLAAAESERFVRQGTGNDEASADVVSLTCPVAAGECAGPADSGDALLERNYPTGAEFLGDGGDVSFSTRGTQNWTVERLLQAHRQLEERGYVFVGYHGTFLEAAQSIVFGGVRARSQDLDAIWRGFYVAGDPALAYGYAQDQEPDARGRIRNGALLRVYVPRSSLPGFYRTGLTLAAPEAAGEVERLIGHPLPLRLDAITGPEEEGGRLETILGWPLAERTVVIPSAIPTDPRNVGGDLAPSSIPDQEQAISALPDYASQPGKPSREDLK

Sequences of produced inactive proteins

TEV-cleavable N-terminal poly-Histidine tag highlighted in grey, with catalytic mutation E>A highlighted in yellow

> *Pseudomonas aeruginosa* exotoxin A (PE)

MHHHHHHENLYFQGAEEAFDLWNECAKACVLDLKDGVRSSRMSVDPAIADTNGQGVLHYSMVLEGGNDALKLAIDNALSITSDGLTIRLEGGVEPNKPVRYSYTRQARGSWSLNWLVPIGHEKPSNIKVFIHELNAGNQLSHMSPIYTIEMGDELLAKLARDATFFVRAHESNEMQPTLAISHAGVSVVMAQAQPRREKRWSEWASGKVLCLLDPLDGVYNYLAQQRCNLDDTWEGKIYRVLAGNPAKHDLDIKPTVISHRLHFPEGGSLAALTAHQACHLPLETFTRHRQPRGWEQLEQCGYPVQRLVALYLAARLSWNQVDQVIRNALASPGSGGDLGEAIREQPEQARLALTLAAAESERFVRQGTGNDEAGAASADVVSLTCPVAAGECAGPADSGDALLERNYPTGAEFLGDGGDISFSTRGTQNWTVERLLQAHRQLEERGYVFVGYHGTFLEAAQSIVFGGVRARSQDLDAIWRGFYIAGDPALAYGYAQDQEPDARGRIRNGALLRVYVPRSSLPGFYRTGLTLAAPEAAGEVERLIGHPLPLRLDAITGPEEEGGRLATILGWPLAERTVVIPSAIPTDPRNVGGDLDPSSIPDKEQAISALPDYASQPGKPPREDLK

> *Vibrio cholerae* Cholix (Chx)

MHHHHHHENLYFQGVEDELNIFDECRSPCSLTPEPGKPIQSKLSIPSDVVLDEGVLYYSMTINDEQNDIKDEDKGESIITIGEFATVRATRHYVNQDAPFGVIHLDITTENGTKTYSYNRKEGEFAINWLVPIGEDSPASIKISVDELDQQRNIIEVPKLYSIDLDNQTLEQWKTQGNVSFSVTRPEHNIAISWPSVSYKAAQKEGSRHKRWAHWHTGLALCWLVPMDAIYNYITQQNCTLGDNWFGGSYETVAGTPKVITVKQGIEQKPVEQRIHFSKGNAMSALAAHRVCGVPLETLARSRKPRDLTDDLSCAYQAQNIVSLFVATRILFSHLDSVFTLNLDEQEPEVAERLSDLRRINENNPGMVTQVLTVARQIYNDYVTHHPGLTPEQTSAGAQAADILSLFCPDADKSCVASNNDQANINIESRSGRSYLPENRAVITPQGVTNWTYQELEATHQALTREGYVFVGYHGTNHVAAQTIVNRIAPVPRGNNTENEEKWGGLYVATHAEVAHGYARIKEGTGEYGLPTRAERDARGVMLRVYIPRASLERFYRTNTPLENAEEHITQVIGHSLPLRNEAFTGPESAGGEDATVIGWDMAIHAVAIPSTIPGNAYEELAIDEEAVAKEQSISTKPPYKERKDELK

> *Aeromonas hydrophila* exotoxin A (Ahx)

MHHHHHHENLYFQGADESFNLWQECATRCTLDLAQGVRASQLDVASLLGGQTDSGVLHYSMVLEEGGDSLKLALGNALTLRTDGTTITLTSATAGKGPRTYSYTRQGRGNWSLHWLVPVGDDAPASIKVFFHELDAGSEVSHISPIYSIEVSDDLLRTMASNSTLFVRHVENNEINRSLTLSAAGVGFVAAPTQHSRQKRWSEWHTGKVLCLLDPLDAVYNYLSQRTCNLGDTWEGKVYRVLAGAPASHDTHIVPTAISHRLHFAKGDGLAALTTHQVCAIPLESLARSRQPRGWEELSQCGYPVHNLVTLYLLTRLPWSQLDTVITQALANTTPEDGSTPRGQLAQAIRENPAQARLALSMAAAQSDAFSHQQAGNSQEQAASADVVNLTCPAADLNCLAPADSADALQERDYPNGDSFLGDGDEVSFSTAGTRNWSVTRLEQVHRQLLARGYLFVGYHGTFLEAAHSIVFEGVHERDQSSIAPWQGFYVAGDPALAYGYAQDQEADARGRIRNGVLLRVYVPRAALPRLYATQQTLADPGAVDEVGRLIGHPLPLQLEAITGPEEEGGRLATILGWRLAEQAVVIPSTIPTDPRNVGGNLDPASVPQEESAISSLPDYTTQPREDLK

> *Chromobacterium haemolyticum* exotoxin A (Hmx)

MHHHHHHENLYFQGKTDFEIFKQCADNCILSPAEPGKFISTSLPLQITPSPDEGVLYYSMFVQDRFAAAANNSATIKIDEFAKVRINDGQGTGHAPGTLTIELATPDGKVKKFTHKRRTEWFTLNWVVPIGKDAPTSIKLFIMDMDSNKKIVDHSPLYSVDLDDAALARWPDKAKLAFSSANPRNDIILSWPGVGYTAAPTQHNRQKRWSEWHSGILLCWLDPLDAIYNYVTQNRCQLNKTWEGKLYQVVAGKPQINEFKPLAKAPIQHRVHFSKENALGALSAHRVCGIPLESLARSRQPRGWEELSACGYRVESIVGLYIATRLSFDRFRQVVDDLIHSRPVSGAQDPEALEQLGTAVRETPGLAREGLAEAEALLDTYLDYHPGASADDAQRADVLSLTCPADSEPCAAANADGAHVNLEYHPGSSFFAPGELVEFLSNGTTSNWSQERLLATHQRLLDQGYVFAGYHGGSTIAARSIVTGGITPRTQELPPIWKGFYIAGNPEVAYGYALDNDNPRSRGIMMRIYVPRTALPQLFRTSQPLSDEAAALREMSRLFGRNVTLDSTLGYESITGPQAPGEADATVLGWLMARHSVAIPSMIQGNGNNAGKIDVPDYEKKISALPDYVTKR

> *Collimonas fungivorans* exotoxin A (Cfx)

MHHHHHHENLYFQGIEGNFRLWDDCSSYCLVYAAPHKIYQTPLATQAVPDSPGKEGVLHYSMVMKDYVGNGQVLALRLDDFATVFVEQESLRLSLLGSDGKPRNFQYARQGAKHWSLNWLVPVGDDAPTSIKVFFKNLDGQNNILSISPLYSVEVDDKTLARWPALATFSVTQENVTQGQGLLGIRRAGVSYVAAPVNHDRHKRWSEWHSGKLLCLLDPLDAIYNYVSQNRCSLGETWEGAIYQTLAGRPVDKYA

PPASKPVISQRIHFAKGNALEALTSHRVCGIPLESLARRRKPRGWEEWSSCGNPAANFVALYIATRLPFDQFRQVIHNLVHGQAVAA
PDPVPLDALRTAVIEQPELARQSIAQAADIFRNYQAANPGASAAAAQQADVLAVTCPADARPCGSGASSGVLVQRENPTGAHFLN
DGELPSFTVQGTQNWNLNRLQAAHLRLQVQGYVFAGYHGTSLEGAQSIVFGGIHNRQQDLEEIWRGLYVAGDPALAYGYAQDAE
GDERGRIRNGTMLRVYVPRSALPRLFATSLPLDHPGASQEVARLIGHPLPLLYESITGPEAAGGNRLATILGWQLAEQAVAIPSMIPT
NSRTVGNPLDPATVTLEEKQISSLPGYATKPAKDDKTEL

> *Shewanella putrefaciens* exotoxin A (Spx)

MHHHHHHENLYFQGVNEAFDLWQECATHCQLDLSQGIRSSELDLTPLFETSNEEGILHYSMLLGEGNEGLKLAIDNALTLHTTHSTI
NFTSETAESGPRSYSYIRKGENNWSLNWLVPVGDDAPASIKIFFLEQDAVGLNRYISPIYSIEVSNNLLNSLAHKSTFYIRAFDNNQTLS
MVNISSAGVSYVAAPQQHHRQKRWSEWHTGKLLCFLDPFDAFYNYVTQHTCNPDDTWEGQIYRVLAGNPATLDTTAPSTTPAVI
SHRIHFDRGNSLASLTAHQVCGIPLESLARTRHPRGWEELNNCGYPVRNLVSLFILARLSWDRVEQVIHNALTNPTPGNALDDAIRE
APERARVTLTLAAAQVNQFDNQAAGNTPEQAQSADVVSLSCSAGALHCSAPADSANALLEREHPNGANFLGAGEAVSFTTRGTR
NWSSARLNHAHQQLIARGYVFVGYHGSSLEGAQSIVFGGIRTRTQALDDVWQGLYISGDPAVAYGYAQDQEPDSRGRIRNGTML
RVYVPGTATAYLYETPLTLADPEAVDAVGHLIGHPLPLQTEAITGPEEAGGRPATILGWELAEQAVAIPSTIPTDPSNIGGDLDPSSIPD
EESDISALPDNVTKPHHDEL

> *Janthinobacterium lividum* exotoxin A (Jlx)

MHHHHHHENLYFQGIKTDFEIFKQCADSCILSPSEPGKFISTSLPVQITPSPDEGVLYYSMFVQDRFASAANNSATIKIDDFAKIRIND
GYDTGSAPGSLTIELATPDGQVKKFTHKRRTEWFTLNWAVPIGKDAPTSIKLFIMDIDSNKKIVDHSPLYSVDLDDAALARWPAKAK
LAFSSANAMNDIILSWPGVGYTAAPTQHSRQKRWSEWHSGILLCWLDPLDAIYNYVTQNRCQLKNTWEGSLYKVVAGNPQINEF
KPVAKAPIQHRVHFSKENALGALSAHRVCGIPLESLARSRQPRGWEELSACGFRVENIVGLYIATRLSFDRFRQVVEDLIHSRPVSGA
QDPVALEQLGSVVRETPELAREGLAEAEARLNAYRANHPGSSADDAQRADVLSLTCPADSAPCNAPDAAGAHVNREYHPGTDYFA
PGEPVEFLPNGTTRNWSQDRLLATHQRLLDLGYVFAGYHGGSIAAARSIVTGGITRRSQDLQPIWRGLYIASDPEVAYGYALDNNTG
ATGTMMRVYVPGTALSRLFRTNQALSDEAAALREIGRLLGRNITLADTLGYESITGPQDPGDPDATILGWDLAEHAVVIPSMIRGN
NGSQLNVPDYEKKISPLPNYVTKR

> *Serratia fonticola* exotoxin A (Sfx)

MHHHHHHENLYFQGANDKFDLWTQCADSCSLDLSNQRIWSSPMDLSLFGQTSEQGVLHFSMVLGENNDSVRIGIDNAFSLLINE
NSIQFKGVSDITKPVKFNYTRQDKGNISVNWLVPIGGGTPSEIKVFIHEVNSANQITSMSPIYTIPVSNEVLSTLEKKSTIYIRGTNSNSL
SYELIVKNAGVSIASTQSGPTRKRRWTHWDSGKTLCFIDPLNAIYNYLSQNTCQLDDTWEGKVYLTLYGSPATHDIEMPASPISTRTH
FSEGGSLAALTAHRVCAIPFETFTRHRQPRGWEDLEKCGYPVHSLIAFYIAARHSWSQVDQVIQNALDNQESDSDLDRAIRESPQQ
SRLALTTAAQTSAVFVSQSSNNTEASAANADILTLTCPLSGLNCMVPADGNEAHQERIYPTGAIFLGDGDDISFTTRGTQNWTTDRL
IAAHQQLTSQGYVFVGYHGTFLEAAHSIVFEGVRARSQSLDNAWHGFYIAGDPAVSYGYALDQDPDPNTGRIRNGVMLRVYVRQ
ESLPNFYQTNVALSSPDAVNAVSSLIGHTLPLQLDSITGQEDEEGRLATIIGWPLAEQLVVIPSTISTDPRNPGGVLDPSSIPEAEKAISV
LPNYSTSPTNTH

> *Acinetobacter baumannii* exotoxin A (Abx)

MHHHHHHENLYFQGAEEAFDLWNECAKACVLDLKDGVRSSRMSVDPAIADTNGQGVLHYSMVLEGGNDALKLAIDNALSITSD
GLTIRLEGGVEPNKPVRYSYTRQARGSWSLNWLVPIGHEKPSNIKVFIHELNAGNQLSHMSPIYTIEMGDELLAKLARDATFFVRAH
ESNEMQPTLAISHAGVSVVMAQAQPRREKRWSEWASGKVLCLLDPLDGVYNYLAQQRCNLDDTWEGKIYRVLAGNPAKHDLDI
KPTVISHRLHFPEGGSLAALTAHQACHLPLETFTRHRQPRGWEQLEQCGYPVQRLVALYLAARLSWNQVDQVIRNALASPGSGGD
LGEAIREQPEQARLALTLAAAESERFVRQGTGNDEASADVVSLTCPVAAGECAGPADSGDALLERNYPTGAEFLGDGGDVSFSTRG
TQNWTVERLLQAHRQLEERGYVFVGYHGTFLEAAQSIVFGGVRARSQDLDAIWRGFYVAGDPALAYGYAQDQEPDARGRIRNGA
LLRVYVPRSSLPGFYRTGLTLAAPEAAGEVERLIGHPLPLRLDAITGPEEEGGRLATILGWPLAERTVVIPSAIPTDPRNVGGDLAPSSI
PDQEQAISALPDYASQPGKPSREDLK

DNA sequences

>ENA|AAB59097|AAB59097.1 Pseudomonas aeruginosa exotoxin type A

ATGCACCTGATACCCCATTGGATCCCCCTGGTCGCCAGCCTCGGCCTGCTCGCCGGCGGCTCGTCCGCGTCCGCCGCCGAGGA
AGCCTTCGACCTCTGGAACGAATGCGCCAAAGCCTGCGTGCTCGACCTCAAGGACGGCGTGCGTTCCAGCCGCATGAGCGTC
GACCCGGCCATCGCCGACACCAACGGCCAGGGCGTGCTGCACTACTCCATGGTCCTGGAGGGCGGCAACGACGCGCTCAAGC
TGGCCATCGACAACGCCCTCAGCATCACCAGCGACGGCCTGACCATCCGCCTCGAAGGCGGCGTCGAGCCGAACAAGCCGGT
GCGCTACAGCTACACGCGCCAGGCGCGCGGCAGTTGGTCGCTGAACTGGCTGGTACCGATCGGCCACGAGAAGCCCTCGAAC
ATCAAGGTGTTCATCCACGAACTGAACGCCGGCAACCAGCTCAGCCACATGTCGCCGATCTACACCATCGAGATGGGCGACGA
GTTGCTGGCGAAGCTGGCGCGCGATGCCACCTTCTTCGTCAGGGCGCACGAGAGCAACGAGATGCAGCCGACGCTCGCCATC
AGCCATGCCGGGGTCAGCGTGGTCATGGCCCAGACCCAGCCGCGCCGGGAAAAGCGCTGGAGCGAATGGGCCAGCGGCAA
GGTGTTGTGCCTGCTCGACCCGCTGGACGGGGTCTACAACTACCTCGCCCAGCAACGCTGCAACCTCGACGATACCTGGGAAG
GCAAGATCTACCGGGTGCTCGCCGGCAACCCGGCGAAGCATGACCTGGACATCAAACCCACGGTCATCAGTCATCGCCTGCAC
TTTCCCGAGGGCGGCAGCCTGGCCGCGCTGACCGCGCACCAGGCTTGCCACCTGCCGCTGGAGACTTTCACCCGTCATCGCC
AGCCGCGCGGCTGGGAACAACTGGAGCAGTGCGGCTATCCGGTGCAGCGGCTGGTCGCCCTCTACCTGGCGGCGCGGCTGTC
GTGGAACCAGGTCGACCAGGTGATCCGCAACGCCCTGGCCAGCCCCGGCAGCGGCGGCGACCTGGGCGAAGCGATCCGCGA
GCAGCCGGAGCAGGCCCGTCTGGCCCTGACCCTGGCCGCCGCCGAGAGCGAGCGCTTCGTCCGGCAGGGCACCGGCAACGA
CGAGGCCGGCGCGGCCAACGCCGACGTGGTGAGCCTGACCTGCCCGGTCGCCGCCGGTGAATGCGCGGGCCCGGCGGACA
GCGGCGACGCCCTGCTGGAGCGCAACTATCCCACTGGCGCGGAGTTCCTCGGCGACGGCGGCGACGTCAGCTTCAGCACCCG
CGGCACGCAGAACTGGACGGTGGAGCGGCTGCTCCAGGCGCACCGCCAACTGGAGGAGCGCGGCTATGTGTTCGTCGGCTA
CCACGGCACCTTCCTCGAAGCGGCGCAAAGCATCGTCTTCGGCGGGGTGCGCGCGCGCAGCCAGGACCTCGACGCGATCTGG
CGCGGTTTCTATATCGCCGGCGATCCGGCGCTGGCCTACGGCTACGCCCAGGACCAGGAACCCGACGCACGCGGCCGGATCC
GCAACGGTGCCCTGCTGCGGGTCTATGTGCCGCGCTCGAGCCTGCCGGGCTTCTACCGCACCAGCCTGACCCTGGCCGCGCCG
GAGGCGGCGGGCGAGGTCGAACGGCTGATCGGCCATCCGCTGCCGCTGCGCCTGGACGCCATCACCGGCCCCGAGGAGGAA
GGCGGGCGCCTGGAGACCATTCTCGGCTGGCCGCTGGCCGAGCGCACCGTGGTGATTCCCTCGGCGATCCCCACCGACCCGC
GCAACGTCGGCGGCGACCTCGACCCGTCCAGCATCCCCGACAAGGAACAGGCGATCAGCGCCCTGCCGGACTACGCCAGCCA
GCCCGGCAAACCGCCGCGCGAGGACCTGAAGTAA

>ENA|AAW80252|AAW80252.1 Vibrio cholerae hypothetical exotoxin A

CTGTACTTGACATTTTATTTGGAGAAAGTAATGAAGAAGATGTTATTGATAGCAGGGGCTACGGTGATATCCAGTATGGCTCATC
CCACATTTGCAGTCGAAGATGAGTTAAACATATTTGATGAATGCCGTTCGCCATGTTCGTTGACCCCGGAACCGGGTAAGCCGA
TTCAATCAAAATTGTCTATCCCTAGTGATGTTGTTTTAGATGAAGGTGTTCTGTATTACTCGATGACGATTAATGATGAGCAGAAT
GATATTAAGGATGAGGACAAAGGAGAGTCCATTATTACTATTGGTGAATTTGCCACAGTAAGAGCGACTAGACATTATGTTAATC
AAGATGCGCCTTTTGGTGTCATTCATTTAGATATTACGACAGAAAATGGTACAAAAACGTACTCTTATAACCGAAAAGAGGGTG
AATTTGCAATTAATTGGTTAGTGCCTATTGGTGAAGATTCTCCTGCAAGCATTAAAATTTCCGTTGATGAGCTCGATCAGCAAAG
AAATATCATCGAGGTGCCTAAACTATATAGTATTGATCTCGATAACCAAACGTTAGAGCAGTGGAAAACCCAAGGTAATGTTTCTT
TTTCGGTAACGCGACCTGAACATAATATTGCTATTTCTTGGCCAAGCGTGAGTTACAAAGCAGCGCAGAAAGAGGGTTCACGC
CATAAGCGTTGGGCTCATTGGCATACAGGATTAGCACTATGTTGGCTTGTGCCAATGGATGCTATTTATAACTATATCACCCAGCA
AAATTGTACTTTAGGGGATAATTGGTTTGGTGGATCTTATGAGACTGTTGCAGGAACTCCGAAGGTGATTACGGTTAAGCAAGG
GATTGAACAAAAGCCAGTTGAGCAGCGCATCCATTTCTCCAAGGGGAATGCGATGAGCGCACTTGCTGCTCATCGAGTCTGTG
GTGTGCCATTAGAAACTTTGGCGCGCAGTCGGAAACCTAGGGATTTAACGGATGATTTATCATGTGCCTATCAAGCGCAGAATA
TTGTGAGTTTATTTGTCGCGACGAGAATTTTATTCTCTCATCTAGATAGCGTATTTACTCTGAATCTTGACGAACAAGAACCAGAG
GTGGCTGAAAGGCTAAGTGATCTTAGACGAATTAATGAAAATAACCCCGGCATGGTTACACAGGTTTTAACCGTTGCTCGACAG
ATCTATAACGATTATGTCACTCACCATCCCGGATTAACTCCTGAGCAAACCAGTGCGGGTGCACAAGCTGCCGATATCCTCTCTTT
ATTTTGCCCAGATGCTGATAAGTCTTGTGTGGCTTCAAACAACGATCAAGCCAATATTAACATTGAGTCTCGTTCTGGCCGTTCAT
ATTTGCCTGAAAACCGTGCGGTAATCACCCCTCAAGGAGTCACAAATTGGACTTACCAGGAACTCGAAGCAACACATCAAGCT

CTGACTCGAGAGGGTTATGTGTTCGTGGGTTACCATGGTACGAATCATGTCGCTGCGCAAACCATAGTGAATAGAATTGCCCCT
GTTCCGAGAGGCAACAACACTGAAAACGAGGAAAAGTGGGGCGGGTTATATGTTGCAACTCACGCTGAAGTTGCCCATGGTT
ATGCTCGGATCAAAGAAGGGACAGGGGAGTATGGACTTCCGACCCGTGCTGAGCGGGACGCTCGTGGGGTAATGCTAAGAGT
GTATATCCCTCGTGCTTCATTAGAACGTTTTTATCGAACGAATACACCTTTGGAAAATGCTGAGGAGCATATAACGCAAGTGATT
GGTCATTCTTTGCCATTACGAAATGAAGCATTTACTGGTCCAGAAAGTGCGGGAGGGGAAGACGAAACTGTCATTGGCTGGG
ATATGGCGATTCATGCAGTTGCGATTCCTTCGACTATTCCAGGGAACGCTTACGAAGAATTGGCGATTGATGAGGAGGCTGTTG
CAAAAGAGCAATCGATTAGCACAAAACCACCTTATAAAGAGCGAAAAGATGAACTGAAATAA

>ENA|AXV34983|AXV34983.1 Aeromonas hydrophila exotoxin

TTGGCCATGCTAGGCGGTGCCATCTCACCGGTCATGGCCGACGAGTCATTCAACCTGTGGCAGGAGTGCGCCACCCGTTGCAC
TCTGGATCTGGCCCAGGGGGTGCGCGCCAGCCAGCTCGATGTCGCCGGCCTGCTGGGTGAACAAGCCGGATCCGGGGTACTG
CACTACTCCATGGTATTGGAGGAAGGAGGAGACAGCCTCAAGCTGGCCCTCGGCAATGCGCTGACCCTGCGCACGGATGGCA
CCACCATCACCCTGACCAGCGCCACCGCGGGCAAGGGCCCGCGCACCTACAGCTATACCCGCCAGGGACACGGCAACTGGTC
GCTGCATTGGCTGGTTCCCGTCGGCGACGATGCGCCCGCCAGCATCAAGGTCTTCTTCCACGAGCTGGATGCAGGATCCGAGG
TGAGCCATATCTCCCCCATCTACAGCATCGAAGTGAGCGACGACCTGCTGCGCAGCATGGCCAGCAACTCGACACTCTTCGTCA
GGCACGTGGAAAACAATCAGATCAATCGCAGCCTCACCCTCAGTGCGGCCGGTGTCGGCTTCGTGGCGGCGCCCACCCAGCA
CAGCCGTCAGAAGCGCTGGAGCGAGTGGCACAGCGGCAAGGTGTTGTGCCTGCTGGATCCGCTGGATGCGGTCTACAACTAC
CTGTCGCAGCGCACCTGCAACCTGGGTGATACCTGGGAAGGCAAGGTCTACCGGGTATTGGCCGGCGCACCCGCCTCTCACG
ATACCCATATCGTGCCGACCGCCATCAGTCATCGTCTGCACTTTGCCAAGGGAGACGGTCTGGCGGCCCTCACCACTCATCAGG
TTTGCGCCATACCGCTTGAATCCCTGGCCAGAAGCCGTCAACCCAGAGGCTGGGAAGAGCTCAGCCAGTGCGGTTATCCGGTC
CACAATCTCATCACCCTCTATCTACTGACCCGCCTGCCATGGTCACAGCTTGATACCGTGATCACCCAGGCGCTGGCCAACACCA
CGCCAGAGGATGGTTCAACGCCACGAGGGCAGCTGGCACAAGCCATTCGGGAGAACCCGGCCCAGGCACGGCTGGCCCTGA
GCATGGCGGCCGCCCAGAGCGACGCCTTCAGCCACCAACAGGCGGGCAATAGCCAAGAACAGGCCGCCAGCGCCGACGTGG
TCAATCTCACCTGCCCTGCCGCCGATCTCAACTGCCTGGCGCCGGCCGACAGCGCCGATGCCCTGCAGGAGCGGGACTATCCG
AACGGTGCCAGCTTCCTGGGTGACGGGGACGAGGTCAGCTTCAGCACGGCCGGTACTCGCAACTGGTCGGTGACCCGCCTG
GAGCAAGCCCATCGCCAGTTGCTGGCGCGCGGTTATCTCTTCGTCGGTTATCACGGCACCTTCCTCGAAGCCGCCCACAGCATA
GTGTTTGAAGGGGTGCATGAACGGGATCAATCCTCCATCGCTCCTTGGCAAGGCTTCTATGTCGCTGGCGATCCGGCCCTCGCC
TACGGTTACGCCCAGGATCAGGAAGCGGATGCCCGTGGCCGCATCCGCAATGGCGTGCTGCTGCGGGTGTATGTGCCGCGCG
CCGCGCTGCCTCACCTCTACGCGACCCAGCAGACCCTGGCCGATCCCGGCGCCGTCGATGAGGTCGGCCGCCTGATTGGTCAC
CCCCTGCCGCTGCAGCTGGAGGCCATCACAGGACCGGAAGAGGAAGGTGGACGCCTGGAAACCATTCTGGGTTGGCGGCTG
GCGGAGCAGGCGGTGGTGATCCCCTCCACCATACCGACGGATCCGCGCAATGTCGGCGGCGATCTTGACCTTGCCAGCGTGCC
GCAGGAAGAGAGCTCCATCAGCGCCTTGCCTGATTACACCACCCAGCCCCGCGAAGACCTCTGA

>MUKV01000035.1:37277-39136 Chromobacterium haemolyticum strain H5244 35, whole genome shotgun sequence [GM note: Reverse complement]

ATGCGATTGTATAAAAGCCTTGCCCTTGCCGCGGGCCTGATTCCCTTATTGTCCGCGTGCGAAACCGACGAAGCCAGCCCCTTT
GCCGCCGGAACGGCACAAACAGCCAGTTTCAAACAAGCCGCCAGTTCCGTCATCAAAACCGATTTCGAAATCTTCAAACAATG
CGTGGACAACTGCATTCTCTCCCCGGCGGAACCGGGTAAATTCATCAGCACCTCATTGCCGCTACAAATCACCCCGTCGCCGGA
CGAAGGCGTGCTGTATTACTCGATGTTCGTGCAAGACCGTTTCACCACCGCCGCCAATAACAGCGCCACCATCAAGATCGACGA
TTTCGCCAAGGTCCGGATCAACGACGGCCAGGGCACCGGTCACGCGCCGGGCACCTTGACCATTGAACTGGCCACCCCGGAC
GGCAAGGTCAAGAAATTCACGCATAAGCGCCGCACTGAATGGTTCACGCTGAATTGGGCGGTGCCCATCGGCAAAGACGCGC
CCACCAGCATCAAGCTGTTCATCATGGACATGGACAGCAATAAGAAGATCGTCGATCACTCGCCACTGTACAGCGTGGACCTGG
ACGACACCGCGCTGGCGCGCTGGCCGGACAAGGCCAAGCTGGCGTTCAGCTCCGCCAATCCGAGGAATGACATCATCCTGTC
TTGGCCGGGCGTGGGCTACACCGCCGCGCCAGCGCAGCACAACCGCCAAAAGCGCTGGAGCGAATGGCATAGCGGCGTCCT
GCTGTGCTGGCTGGACCCGCTGGACGCCATCTACAACTACGTCACCCAAAACCGCTGCCAGTTGAACAAGACCTGGGAAGGC

GCGCTGTACAAGGTGGTGGCCGGCAAGCCGCAGATCAATGAATTCAAGCCGCTGGCCAAAGCGCCCATCCAGCACCGGGTGC
ACTTCAGCAAGGAAAACGCACTGGGCGCGCTGTCCGCCCACCGCGTCTGCGGCATCCCGCTGGAGTCGCTGGCCCGCAGCCG
CCAGCCCAGGGGCTGGGAAGAGCTGTCCGCTTGCGGCTTCCGGGTGGAAAACATCGTCGGGCTATACATCGCCACCCGGCTG
TCCTTCGACCGTTTCCGCCAGGTGGTCGATGACTTGATCCATTCCCGCCCGGTCAGCGGCGCGCAAGACCCGGTGGCGCTGGA
GCAATTGGGCACCGCGGTGCGCGAAACGCCGGGACTCGCCCGCGAAGGCCTGGCCGAGGCGGAGGCCCTTCTGGACACCTA
CCTCGACTATCATCCTGGCGCCAGCGCCGATGACGCGCAAAGAGCGGACGTGCTGTCGCTGACGTGCCCGGCCGACAGCGAG
CCTTGCGCCGCCGCCAACGCCGACGGCGCCCACGTCAACCTCGAGTACCACCCCGGCTCAAGCTTCTTCGCGCCGGGCGAGC
CGGTGGAATTCCTCGCCAACGGCACCACCCGCAACTGGAGCCAGGAGCGCCTGCTGGCCACCCATCAGCGGCTGCTGGACCA
GGGCTATGTCTTCGCCGGCTATCACGGCGGCTCAACCATCGCAGCGAGAAGCATCGTCACCGGCGGCATCACGCCCCGCACCC
AGGAGCTACCACCGATCTGGAAGGGCTTCTACATCGCCGGCAATCCGGAAGTCGCTTACGGCTACGCGCTGGACAACGACAAC
CCCCGCTCTCGCGGCATCATGATGCGCATCTACGTGCCTAGAACCGCTCTACCCCAATTGTTCCGCACCAGCCAGCCGCTCAGCG
ACGAGGCCGCGGCGCTCAGGGAGATGAGCCGCCTGTTTGGCCGCAACGTCACACTGGATAGTACCTTAGGATATGAATCCATC
ACCGGGCCACAAGCTCCCGACGAGGCGGATGAAACCGTCCTGGGTTGGCTTATGGCGCGACATTCAGTGGCGATTCCGTCGA
TGATTCAGGGCAACGGCAACAACGCCGGGAAGATCGACGTGCCGGACTACGAGAAGAAGATCAGCGCTCTGCCCGACTACGT
CACTAAGCGCTG

>NZ_VOQB01000003.1:c67414-65456 Shewanella sp. YLB-07 NODE_3_length_628018_cov_39.817077, whole genome shotgun sequence

ATGATAAAAACACGATATCTCTGCTTGAGTGTTGCGATTAGCTCACTGTTTCTCGCAATAACTAGCGTAAATGCAACGGATCTGC
GTGGCTTAGAACCACAGATAACCTCAGCAGCTCAAGTCAATGAAGCATTCGATCTATGGCAAGAATGTGCTACTCACTGCCAAC
TCGATTTAAGCCAAGGTATTAGATCAAGTGAATTGGATCTGACTCCCTTATTTGAAACCAGTAATGAAGAAGGCATACTGCACTA
CTCTATGTTACTGGGAGAAGGTAATGAGGGGCTCAAACTTGCTATCGATAATGCTCTGACACTACATACTACTCATTCAACGATTA
ATTTTACCAGTGAGACGGCGGAATCCGGTCCTCGCAGTTACTCATATATTCGTAAGGGAGAAAATAATTGGTCTCTAAACTGGCT
TGTTCCCGTTGGTGATGATGCTCCAGCCAGCATTAAAATATTCTTCCTTGAACAGGACGCCGTAGGCCTGAACAGATATATCTCTC
CCATCTACAGTATAGAGGTGAGTAACAACCTGTTAAATAGTTTGGCGCATAAATCCACGTTTTATATTAGGGCTTTCGACAATAAC
CAAACATTGTCAATGGTCAATATCAGCAGTGCCGGAGTGAGCTATGTGGCAGCGCCGCAGCAGCACCATCGACAGAAACGCTG
GAGTGAATGGCACACAGGTAAATTACTCTGCTTCCTTGACCCTTTCGATGCGTTCTACAATTACGTGACTCAACACACCTGTAAT
CCTGATGACACGTGGGAAGGGCAAATTTATCGCGTGTTGGCAGGTAATCCAGCGACCCTGGATACAACCGCTCCCTCAACGAC
ACCGGCCGTTATCAGTCATCGAATCCATTTCGATAGAGGTAACAGCCTAGCATCCTTAACTGCCCATCAGGTATGCGGTATTCCTC
TTGAATCTCTAGCTCGTACCCGCCATCCCAGAGGTTGGGAAGAACTCAATAATTGTGGTTATCCGGTACGTAACCTTGTCAGCCT
CTTTATATTGGCCCGACTATCATGGGACAGGGTAGAGCAAGTGATCCACAATGCTTTGACCAACCCGACTCCTGGTAATGCGCT
GGATGATGCTATTCGAGAAGCACCAGAGAGAGCTAGGGTGACCCTGACCCTGGCAGCTGCGCAGGTCAATCAATTTGATAATC
AGGCCGCTGGCAATACGCCTGAACAAGCCCAAAGTGCTGACGTGGTGAGCCTATCCTGCTCTGCAGGTGCGCTTCATTGTAGT
GCGCCAGCCGACAGTGCCAACGCGTTACTCGAACGAGAACACCCCAATGGTGCAAACTTTCTAGGAGCGGGGGAGGCGGTA
AGCTTTACCACGAGGGGAACCAGAAACTGGTCTTCAGCACGCTTGAATCATGCACATCAGCAACTGATCGCGCGAGGGTATGT
TTTTGTGGGCTATCACGGCAGCTCTTTGGAAGGTGCTCAAAGCATTGTTTTCGGGGGGATCAGAACCAGAACACAGGCACTG
GATGATGTATGGCAAGGCTTGTATATATCAGGTGACCCTGCCGTCGCCTATGGATATGCGCAAGATCAAGAGCCTGACTCGCGTG
GCCGGATCCGCAATGGCACTATGTTGAGAGTCTATGTACCAGGCACTGCCACAGCATACCTCTATGAAACGCCTCTGACTCTGGC
CGATCCTGAAGCTGTCGATGCTGTAGGTCACCTGATTGGCCACCCACTTCCGTTACAAACGGAAGCCATCACTGGCCCCGAAGA
AGCAGGCGGGCGCCCGGAAACCATTCTCGGCTGGGAACTGGCTGAACAGGCTGTAGCAATCCCCTCCACGATACCGACAGAT
CCGAGTAATATCGGCGGTGATCTCGACCCATCGAGTATTCCTGATGAGGAAAGTGATATCAGTGCATTACCAGACAATGTCACTA
AACCTCATCACGACGAATTATAG

>PDBX01000002.1:22019-23977 Collimonas sp. PA-H2 Ga0114272_12, whole genome shotgun sequence

ATGTCCAAATTCATGTCGCAACGCTGGATCTATCTGTTCGGATGGCTTTTCCTGCTCGGAACCGGCAGCACCAGGGCGAATACG
ATAGAGGGAAACTTCAGGCTTTGGGACGATTGCAGCAGCTACTGCCTGGTTTACGCCGCCCCCCATAAAATCTATCAGACCCCT
TTGGCCACGCAGGCTGTGCCGGATTCTCCCGGCAAGGAAGGCGTACTGCATTACTCCATGGTGATGAAAGACTATGTCGGCAA
CGGCCAGGTTCTGGCCTTGCGCCTTGACGATTTCGCCACCGTCTTTGTCGAGCAAGAAAGCCTGAGACTGAGCCTGCTTGGCT
CTGACGGCAAGCCCAGGAATTTCCAGTATGCTCGCCAAGGCGCGAAACATTGGTCGCTTAACTGGCTGGTGCCCGTTGGCGAT
GACGCTCCCACCAGCATCAAGGTCTTCTTCAAGAACCTCGACGGCCAGAATAATATTCTCTCCATCTCGCCTCTTTACAGTGTGG
AGGTAGACGACAAGACACTCGCGCGCTGGCCCGCACTCGCCACCTTCTCTGTCACCCAGGAGAATGTCACTCAGGGCCAAGG
CCTGCTTGGGATACGACGTGCAGGCGTCAGTTACGTCGCCGCTCCCGTGAACCATGACCGTCACAAACGCTGGAGCGAGTGG
CACAGCGGCAAGCTGCTCTGCCTGCTCGACCCTTTGGATGCCATTTACAACTACGTCAGCCAGAACCGCTGCAGTCTGGGCGA
AACCTGGGAAGGCGCAATTTATCAAACCCTGGCAGGCCGGCCTGTCGACAAATACGCCCCACCGGCAAGCAAGCCAGTTATTA
GCCAACGCATCCATTTTGCCAAGGGCAACGCTCTGGAAGCGCTAACCAGTCACCGGGTATGCGGCATACCTCTTGAGTCCTTG
GCACGCAGACGCAAGCCGAGAGGATGGGAAGAATGGTCTTCGTGTGGCAATCCCGCGGCCAATTTCGTAGCCCTTTACATCGC
CACCCGCTTGCCGTTCGACCAATTCCGTCAGGTGATTCATAACCTGGTTCACGGGCAAGCAGTTGCAGCGCCTGATCCGGTGCC
TCTGGATGCCCTGAGAACGGCCGTCATTGAGCAACCGGAACTGGCACGTCAAAGCATCGCCCAGGCAGCCGACATCTTCAGG
AACTACCAGGCAGCCAATCCTGGCGCCAGTGCCGCCGCCGCACAACAGGCCGATGTGCTGGCCGTCACCTGTCCGGCTGACG
CGCGCCCGTGCGGCTCCGGCGCAAGCAGCGGCGTGCTGGTCCAGCGTGAAAATCCCACCGGAGCGCACTTTCTGAATGACGG
AGAGCTGCCAAGCTTCACCGTACAAGGCACGCAAAACTGGAACCTGAACCGCCTGCAGGCTGCTCACCTGCGCCTGCAGGTG
CAGGGATACGTTTTTGCGGGTTATCACGGCACATCGCTGGAGGGCGCACAGAGTATCGTGTTTGGCGGCATCCATAACCGCCA
GCAGGATCTTGAGGAAATCTGGAGAGGCCTGTACGTTGCCGGAGATCCCGCTCTTGCCTACGGCTATGCGCAAGATGCGGAAG
GAGATGAGCGCGGCCGCATCCGCAACGGCACGATGTTGCGCGTCTATGTCCCGCGCAGCGCTCTCCCGCGACTCTTTGCCACC
AGCCTGCCTCTGGACCATCCAGGCGCCTCGCAAGAAGTTGCGCGTTTGATCGGCCACCCGCTGCCCTTGCTTTATGAATCGATC
ACCGGTCCGGAAGCAGCAGGCGGCAATCGCCTTGAGACGATCCTGGGCTGGCAGCTCGCGGAGCAGGCAGTCGCGATTCCC
TCGATGATCCCGACCAATAGCCGCACTGTCGGAAATCCCCTCGATCCCGCCACCGTCACGCTTGAAGAAAAGCAGATCAGCAG
CTTGCCGGGCTACGCCACAAAACCTGCGAAAGACGACAAGACGGAACTATGA

>CP014222.1:847765-849768 Janthinobacterium sp. B9-8, complete genome

GCGCTTGGTGACGTAGTTGGGCAGTGGGCTGATCTTTTTCTCGTAGTCCGGCACATTGAGTTGTGAGCCGTTATTGCCCCGAAT
CATTGACGGAATTACCACCGCATGTTCCGCCAGATCCCAACCCAAGATGGTTTCATCCGGGTCGCCGGGATCTTGCGGCCCGGT
GATCGATTCATATCCCAAGGTATCTGCAAGCGTAATATTTCGGCCGAGCAAGCGACCAATTTCTCTGAGCGCTGCGGCCTCGTCG
CTCAGTGCCTGGTTGGTGCGGAATAACCGGGAGAGAGCGGTGCCGGGTACATAGACGCGCATCATGGTGCCCGTTGCGCCGG
TATTGTTATCCAGGGCGTAGCCGTAGGCGACTTCCGGATCGCTGGCGATGTAGAGGCCGCGCCAGATTGGCTGCAGGTCCTGA
GACCGGCGAGTGATGCCGCCGGTCACAATGCTTCTGGCGGCAGCGATCGAACCGCCGTGATAGCCGGCGAAGACATAGCCCA
GATCCAATAGCCGCTGATGGGTGGCCAGCAGGCGGTCCTGGCTCCAGTTGCGGGTGGTGCCATTAGGGAGGAATTCCACTGG
CTCGCCCGGTGCAAAATAGTCTGTGCCGGGGTGGTACTCGCGGTTGACGTGCGCGCCGGCGGCATCGGGTGCGTTGCAAGGT
GCGCTGTCGGCCGGGCAAGTTAAGGACAGCACGTCCGCTCTTTGCGCGTCATCGGCACTGCTGCCCGGGTGGTTGGCGCGGT
AGGCGTTCAAGCGGGCTTCTGCCTCGGCCAACCCTTCGCGGGCCAGTTCCGGCGTTTCACGCACTACGCTTCCCAATTGCTCC
AGCGCCACGGGGTCTTGAGCACCGCTGACTGGGCGGGAATGGATCAAATCCTCGACCACCTGGCGGAAGCGATCGAAAGAC
AGCCGGGTAGCAATGTACAGGCCGACGATGTTTTCCACCCGGAAGCCGCAGGCAGATAGCTCTTCCCAACCCCTGGGTTGGCG
ACTGCGGGCTAGCGATTCCAGCGGGATGCCGCAGACACGGTGCGCGGATAGCGCGCCGAGTGCGTTTTCCTTGCTGAAGTGC
ACCCGGTGCTGGATCGGCGCTTTGGCTACCGGCTTGAATTCATTGATCTGGGGATTGCCAGCCACCACCTTGTATAGCGAGCCC
TCCCAAGTATTTTTCAGTTGACAGCGATTTTGGGTGACGTAGTTGTAGATGGCGTCTAGCGGGTCCAGCCAGCACAGCAGGAT
GCCGCTATGCCATTCGCTCCAGCGCTTTTGCCGGCTGTGCTGGGTCGGCGCGGCGGTATAGCCCACGCCCGGCCAAGACAGGA
TGATGTCGTTCATGGCATTGGCGGAGCTGAACGCCAGCTTAGCCTTGGCTGGCCAGCGCGCTAGGGCGGCATCGTCCAAGTCC
ACGCTGTATAGCGGCGAGTGATCGACAATCTTTTTATTGCTGTCGATGTCCATGATAAATAACTTGATGCTGGTGGGCGCGTCTT
TGCCAATCGGCACGGCCCAATTCAGGGTGAACCATTCGGTGCGGCGTTTATGAGTAAATTTTTTGACCTGGCCATCGGGTGTG
GCTAATTCGATGGTTAAACTGCCCGGCGCGGAGCCGGTGTCGTAACCATCGTTGATCCGAATCTTGGCGAAGTCGTCAATTTTG
ATGGTGGCGCTATTATTGGCGGCGGAGGCGAAACGGTCTTGTACAAACATCGAATAATATAAAACGCCCTCATCCGGTGATGGG

GTGATTTGCACCGGTAGCGAGGTGCTGATGAACTTGCCGGGTTCGGAGGGGGAGAGTATGCAGCTGTCGGCGCATTGCTTGA
ATATTTCGAAATCGGTCTTGATGATGGAGCTTGCTGCCTGTTGGAAACCGGCGGCAGCCACTGTATTAGATGGACGAACCTCCT
CGGTTGCAGATGGACGGACCTCCTCGGTTACGCATCCGGACAATAATGGAATGAGCCCCGCAGCAATGGCAAACCCTTTATAA
AGTCTCATCAT

>CP050172.1:c68807-66903 Serratia fonticola strain CPSE11 plasmid plas1, complete sequence

ATGTCAAATAAAGCCAAATTAAGGACTGCTGCGATAGCGATTATTCTCGCATCATATTTATCCCTCCCTGCTTTTGCCAACGACAA
GTTTGATTTGTGGACCCAGTGTGCAGATTCTTGCTCACTTGATCTAAGTAACCAAAGAATCTGGTCAAGCCCGATGGACTTATCA
TTATTTGGCCAGACGTCCGAACAAGGTGTACTTCATTTCTCCATGGTACTTGGTGAGAACAATGACTCTGTGCGGATAGGAATA
GACAATGCATTTTCTCTTCTGATAAACGAAAACAGCATTCAGTTCAAAGGTGTCTCTGATATAACTAAACCGGTAAAATTTAATTA
CACCAGACAAGACAAGGGCAATATCTCAGTAAACTGGCTAGTACCCATAGGAGGAGGTACTCCCTCAGAGATAAAAGTTTTCAT
TCATGAGGTCAACTCAGCTAACCAAATCACCTCAATGTCCCCCATATACACAATCCCCGTCTCAAATGAAGTACTCAGCACACTTG
AAAAAAAAATCGACTATATACATTAGAGGGACTAACAGCAACAGCCTATCGTATGAACTTATAGTAAAGAATGCCGGCGTAAGCAT
TGCGAGCACCCAAAGTGGACCTACACGAAAAAGACGCTGGACACACTGGGACAGTGGAAAAACTCTTTGCTTCATTGACCCG
CTAAACGCTATATATAACTACTTATCACAGAATACATGCCAATTAGATGACACTTGGGAAGGTAAAGTTTATCTCACACTTTATGGC
TCTCCCGCTACGCATGATATAGAAATGCCTGCTTCGCCAATCAGCACCAGAACTCATTTCTCGGAAGGGGGATCATTGGCAGCG
CTTACTGCTCATAGAGTATGTGCAATCCCGTTCGAGACTTTCACTCGGCATCGGCAGCCCAGAGGATGGGAAGATTTAGAGAAA
TGTGGTTATCCAGTTCATAGTCTCATTGCCTTTTATATAGCGGCTCGCCATTCCTGGAGCCAAGTGGATCAAGTAATTCAGAACGC
TCTCGATAACCAAGAAAGCGATAGCGATCTAGACCGCGCTATTCGAGAGAGTCCGCAACAATCGCGACTAGCTTTGACTACGGC
AGCTCAAACTAGCGCAGTATTCGTCAGTCAAAGCTCAAACAACACCGAAGCTTCCGCTGCCAATGCAGATATACTCACGCTAAC
CTGCCCACTGTCGGGTTTGAATTGTATGGTGCCCGCAGATGGCAACGAGGCTCACCAAGAAAGAATCTACCCAACGGGGGCTA
TATTCTTGGGGGACGGAGATGATATCAGTTTTACTACCAGGGGCACGCAGAATTGGACAACTGATAGACTTATTGCAGCCCATC
AGCAACTGACTTCACAGGGGTATGTCTTTGTTGGATATCATGGAACTTTTTTAGAAGCCGCCCATAGCATCGTATTCGAAGGAGT
TAGAGCTAGATCACAAAGTCTGGATAACGCCTGGCACGGCTTCTATATTGCCGGCGATCCTGCAGTATCTTATGGTTATGCCCTTG
ATCAAGATCCTGACCCCAATACTGGCCGTATTCGTAATGGAGTTATGCTCCGTGTCTACGTTAGACAAGAATCACTGCCTAACTTT
TACCAAACTAACGTCGCACTATCTTCCCCGGATGCGGTAAATGCTGTATCTAGTTTGATAGGACATACTTTACCGCTTCAACTTGA
TTCTATTACAGGTCAGGAGGACGAGGAAGGACGGTTGGAAACAATCATCGGCTGGCCATTGGCAGAGCAACTTGTAGTAATCC
CATCGACAATCTCTACTGACCCTCGTAACCCTGGGGGAGTTTTAGACCCATCCTCTATCCCTGAAGCAGAGAAGGCGATTAGCG
TTTTGCCAAACTACTCAACGTCACCTACGAATACCCACTAA

>FMVV01000003.1:c239527-237620 Acinetobacter baumannii isolate AB32_M genome assembly, contig: gnlProkkacontig000003, whole genome shotgun sequence
ATGCACCTGACACCCCATTGGATCCCCCTGGTCGCCAGCCTCGGCCTGCTCGCCGGCGGCTTGTCCGCGTCCGCCGCCGAGG
AAGCCTTCGACCTCTGGAACGAATGCGCCAAGGCCTGCGTGCTCGACCTCAAGGACGGCGTGCGTTCCAGCCGCATGAGCG
TCGACCCGGCCATCGCCGACACCAACGGCCAGGGCGTGCTGCACTACTCCATGGTCCTGGAGGGCGGCAACGACGCGCTCA
AGCTGGCCATCGACAACGCCCTCAGCATCACCAGCGACGGCCTGACCATCCGCCTCGAAGGCGGCGTCGAGCCGAACAAGC
CGGTGCGCTACAGCTACACGCGCCAGGCGCGCGGCAGTTGGTCGCTGAACTGGCTGGTACCGATCGGCCACGAGAAGCCCT
CGAACATCAAGGTGTTCATCCACGAACTGAACGCCGGTAACCAGCTCAGCCACATGTCGCCGATCTACACCATCGAGATGGG
CGACGAGTTGCTGGCGAAGCTGGCGCGCGATGCCACCTTCTTCGTCAGGGCGCACGAGAGCAACGAGATGCAGCCGACGC
TCGCCATCAGCCATGCCGGGGTCAGCGTGGTCATGGCCCAGGCCCAGCCGCGCCGGGAAAAGCGCTGGAGCGAATGGGCC
AGCGGCAAGGTGTTGTGCCTGCTCGACCCGCTGGACGGGGTCTACAACTACCTCGCCCAGCAGCGCTGCAACCTCGACGAT
ACCTGGGAAGGCAAGATCTACCGGGTGCTCGCCGGCAACCCGGCGAAGCATGACCTGGACATCAAGCCCACGGTCATCAGT
CATCGCCTGCATTTCCCCGAGGGCGGCAGCCTGGCCGCGCTGACCGCGCACCAGGCTTGCCACCTGCCGCTGGAGACTTTCA
CCCGTCATCGCCAGCCGCGCGGCTGGGAACAACTGGAGCAGTGCGGCTATCCGGTGCAGCGGCTGGTCGCCCTCTACCTGG
CGGCGCGGCTGTCGTGGAACCAGGTCGACCAGGTGATCCGCAACGCCCTGGCCAGCCCCGGCAGCGGCGGCGACCTGGGC
GAAGCGATCCGCGAGCAGCCGGAGCAGGCCCGTCTGGCCCTGACCCTGGCCGCCGCCGAGAGCGAGCGCTTCGTCCGGCA

GGGCACCGGCAACGACGAGGCCAGCGCCGACGTGGTGAGCCTGACCTGCCCGGTCGCCGCCGGTGAATGCGCGGGCCCGGCGGACAGCGGCGACGCCCTGCTGGAGCGCAACTATCCCACTGGCGCGGAGTTCCTCGGCGACGGCGGCGACGTCAGCTTCAGCACCCGCGGCACGCAGAACTGGACGGTGGAGCGGCTGCTCCAGGCGCACCGCCAACTGGAGGAGCGCGGCTATGTGTTCGTCGGCTACCACGGCACCTTCCTCGAAGCGGCGCAAAGCATCGTCTTCGGCGGGGTGCGCGCGCGCAGCCAGGACCTCGACGCGATCTGGCGCGGTTTCTATGTCGCCGGCGATCCGGCGCTGGCCTACGGCTACGCCCAGGACCAGGAACCCGACGCGCGCGGCCGGATCCGCAACGGTGCCCTGCTGCGGGTCTATGTGCCGCGCTCGAGTCTGCCGGGCTTCTACCGCACCGGCCTGACCCTGGCCGCGCCGGAGGCGGCGGGCGAGGTCGAACGGCTGATCGGCCATCCGCTGCCGCTGCGCCTGGACGCCATCACCGGTCCCGAGGAGGAAGGCGGGCGCCTGGAGACCATTCTCGGCTGGCCGCTGGCCGAGCGCACCGTGGTGATTCCCTCGGCGATCCCCACCGACCCACGCAACGTCGGCGGCGACCTCGCCCCGTCCAGCATCCCCGACCAGGAACAGGCGATCAGCGCCCTGCCGGACTACGCCAGCCAGCCCGGCAAACCGTCGCGCGAGGACCTGAAGTAG
