# Supplementary material for: Discovery of mono-ADP ribosylating toxins with high structural homology to Pseudomonas exotoxin A
Source: Commun Biol. 2025 Mar 11;8:413. doi: 10.1038/s42003-025-07845-y (PMC11897225; doi:10.1038/s42003-025-07845-y)
Supplement: Supplementary file 2 — Description of Additional Supplementary Files [file 42003_2025_7845_MOESM2_ESM.docx]

Description of Additional Supplementary Files

**File name:** Supplementary Data 1

**Description:** Raw data from PLT transport experiment associated with Figure 3
